# Effect of pre-farrowing hygiene routine (sub-standard vs. optimal) and creep feeding regime (dry pelleted starter diet vs. liquid mixture of milk replacer and starter diet) on post-weaning intestinal parameters and growth to slaughter in pigs

**DOI:** 10.1093/jas/skae380

**Published:** 2024-12-18

**Authors:** Shiv R Vasa, Gillian E Gardiner, Paul Cormican, Keelin O’Driscoll, Giuseppe Bee, Peadar G Lawlor

**Affiliations:** Pig Development Department, Teagasc, Animal and Grassland Research and Innovation Centre, Moorepark, Fermoy, Co. Cork P61 C996, Ireland; Department of Science, Eco-Innovation Research Centre, South East Technological University, Waterford City, Co. Waterford X91 K0EK, Ireland; Department of Science, Eco-Innovation Research Centre, South East Technological University, Waterford City, Co. Waterford X91 K0EK, Ireland; Teagasc, Animal and Grassland Research and Innovation Centre, Grange, Dunsany, Co. Meath C15 PW93, Ireland; Pig Development Department, Teagasc, Animal and Grassland Research and Innovation Centre, Moorepark, Fermoy, Co. Cork P61 C996, Ireland; Swine Research Unit, Agroscope, Posieux 1725, Switzerland; Pig Development Department, Teagasc, Animal and Grassland Research and Innovation Centre, Moorepark, Fermoy, Co. Cork P61 C996, Ireland

**Keywords:** antibiotic usage, enzyme activity, large litters, microbiome, villus height

## Abstract

The objective was to evaluate the effect of providing a dry pelleted starter diet (DPS) or a liquid mixture of milk replacer and starter diet (LMR + S) to suckling pigs housed in farrowing pens of sub-standard or optimal hygiene conditions on pig growth to slaughter, and post-weaning (PW) intestinal parameters. On day (d) 107 of gestation, 87 sows were randomly allocated to one of four treatments in a 2 × 2 factorial arrangement. The factors were creep feeding (DPS or LMR + S) and pre-farrowing hygiene routine (SUB-STANDARD or OPTIMAL). Pigs were provided with DPS (manually) from d 11 to weaning (at d 28 ± 1.2 of age) or LMR + S using an automatic liquid feeding system from d 4 to weaning. The SUB-STANDARD hygiene routine (pens washed and dried for ~18 h, sows not washed or disinfected) and the OPTIMAL hygiene routine (pens pre-soaked, detergent applied, washed, dried for 3 days, chlorocresol-based disinfectant applied, dried for 3 more days, and sows washed and disinfected with Virkon) were used to obtain SUB-STANDARD or OPTIMAL hygiene conditions, respectively, in farrowing rooms prior to entry of sows. Microbiome analysis was performed on fecal samples from eight focal pigs per treatment, before weaning and at d 21 and d 114 PW. On d 4 PW, 10 pigs per treatment were euthanized to collect intestinal tissue and digesta samples for histological, enzyme activity, and microbiome analysis. Feeding LMR + S to pigs born into the OPTIMAL hygiene increased total dry matter intake compared to all of the other groups (*P* ≤ 0.05) and increased weaning weight compared to DPS feeding under both OPTIMAL and SUB-STANDARD hygiene conditions (*P* ≤ 0.05). Pigs from OPTIMAL farrowing pens had lower clinical cases of disease, diarrhea prevalence, and were slaughtered 3.8 days earlier than those from SUB-STANDARD farrowing pens (*P *≤ 0.05). Suckling piglet mortality was reduced with LMR + S (*P* ≤ 0.05). On d 4 PW, jejunal and ileal villus height were increased by OPTIMAL hygiene and ileal sucrase activity was increased by LMR + S (*P* ≤ 0.05). On d 4 PW, LMR + S-fed pigs from OPTIMAL farrowing pens had a lower relative abundance of *Clostridium_P* in the jejunum. In conclusion, the OPTIMAL hygiene routine increased pre-weaning LMR + S feed intake, reduced clinical cases of disease, improved intestinal structure, and reduced the weaning to slaughter duration, while LMR + S feeding increased weaning weight, intestinal maturity, and reduced pre-weaning mortality.

## Teaser

Creep feeding a liquid mixture of starter diet and milk replacer in an optimal hygiene conditions increased feed intake and weaning weight of suckling piglets. This liquid creep feeding strategy reduced pre-weaning mortality and optimal hygiene reduced the weaning to slaughter duration.

## Introduction

Litter size in sows has increased in recent decades; for example, the number of piglets born alive in Ireland increased from 12.6 to 14.8 between 2012 and 2022 (Teagasc, 2012, 2022). However, sow colostrum and milk yield have not increased accordingly (Farmer, 2022) and therefore, the milk intake per pig in these large litters is reduced, thereby limiting the growth of suckling piglets (Tokach et al., 2020). Furthermore, on commercial farms, piglets are weaned earlier (at 3 to 4 weeks of age) than in the wild and experience an abrupt change in diet from the liquid sow’s milk to a plant-based dry diet, while experiencing a multitude of social and environmental stressors (Lawlor et al., 2020). This leads to a decline in feed intake of piglets in the early post-weaning (PW) period, along with reduced growth and disruption of small intestinal structure and function (Dong and Pluske, 2007).

Supplementing suckling piglets with liquid milk replacer increases nutrient intake and weaning weight (Arnaud et al., 2024). However, providing liquid milk replacer throughout lactation does not expose suckling piglets to plant-based ingredients (commonly found in PW diets) and is not economically viable due to the high cost of milk replacer. Therefore, creep feeding with a dry pelleted diet is more commonly practiced on commercial farms to promote intestinal maturity and facilitate a smooth transition PW (Luo et al., 2022). However, the benefits of dry creep feeding are inconsistent. While some studies showed that dry creep feeding increased weaning weight (Pajor et al., 1991; Arnaud et al., 2024) and feed intake early PW (Muns and Magowan, 2018), other studies did not observe any positive effects (Sulabo et al., 2010a; Christensen and Huber, 2021). This inconsistency is thought to be largely due to variation in creep feed intake at both individual and litter levels (Bruininx et al., 2002; Tokach et al., 2020). Therefore, providing a liquid mixture of milk replacer and starter diet to suckling pigs holds promise with regard to increasing pre-weaning feed intake and growth, while providing exposure to plant-based ingredients prior to weaning. Moreover, mixing starter diet with milk replacer increases the economic viability of the practice compared to supplementing only milk replacer throughout lactation (Arnaud et al., 2024).

The pre-farrowing hygiene routine can also influence pre-weaning growth. Two recent studies, including one from our group, reported increased piglet weaning weight when optimized hygiene routines were implemented in farrowing rooms (Law et al., 2021; Halpin et al., 2024). Halpin et al. (2024) reported lower total bacterial and *Enterobacteriaceae* counts in the pen environment, higher weaning weight and reduced clinical cases of disease, diarrhea prevalence, and medication usage in piglets when an optimized hygiene routine was used. This suggests that piglets reared in optimal hygiene conditions experience fewer infections and therefore less immune stimulation, likely leading to the increased growth rate observed, as raising an immune response utilizes energy and nutrients, reducing their availability for pig growth (Pluske et al., 2018). While an investigation of creep feed intake was not conducted in these studies, it can be speculated that the observed growth improvement was also associated with increased creep feed intake, as immune stimulation is known to reduce feed intake in pigs (Johnson and von Borell, 1994; Johnson, 1998; Dantzer, 2004).

The hypothesis of the current study is that providing a liquid mixture of milk replacer and starter diet in optimal hygiene conditions will increase the dry matter (DM) intake of creep feed, and growth in pigs while improving PW small intestinal structure and function. This study tests this hypothesis in a 2 × 2 factorial arrangement with two factors for pre-farrowing hygiene routine (sub-standard vs. optimal) and two factors for creep feeding regime (a standard manually delivered dry pelleted starter diet (DPS) regime from day 11 post-farrowing vs. a novel regime involving automated delivery of liquid mixture of milk replacer and starter diet from day 4 post-farrowing). The novelty of this study is that it examines the effect of an optimized creep feeding regime in farrowing accommodation with a sub-standard and an optimal hygiene routine.

## Materials and Methods

This study was conducted between June 2022 and March 2023 at the Teagasc Pig Development Department, Moorepark, Fermoy, Co. Cork, Ireland, in accordance with the legislation for commercial pig production set out in the European Communities (Welfare of Farmed Animals) regulations 2010 and in Irish legislation (SI no. 311/2010). The care and use of the animals were approved by the Teagasc Animal Ethics Committee (Approval No. TAEC2021-310) and the Waterford Institute of Technology Ethics Committee (Approval No. WIT2022REC057) and licensable procedures performed on the animals were authorized by the Irish Health Products Regulatory Authority (project authorization no. AE19132/P147).

### Experimental design

The experiment was conducted using four farrowing groups and involved 87 sows (Large White × Landrace, PIC, Hermitage Genetics, Sion Road, Ireland). Sows were artificially inseminated at the onset of standing estrus and again after 24 h using pooled semen (Topigs Norsvin Tempo, Premier Pig Genetics Limited, Ireland). Between 3 and 6 days after service, the sows were introduced to a dynamic group of ~120 gestating sows. On day 107 of gestation, the sows were weighed and blocked on the basis of parity group (mean ± SD; 2.4 ± 1.68), number of pigs weaned at the previous farrowing (13.4 ± 2.28 for multiparous sows), and bodyweight (278.1 ± 30.63 kg) into 22 blocks of four sows. The distribution of sow parity group was as follows: group 1, parity 0 (13%); group 2, parity 1 and 2 (41%); and group 3, parity 3 to 6 (46%). On day 110 of gestation, the sows were transferred to standard farrowing rooms with 14 farrowing pens per room. Within block, sows were randomly allocated to one of four treatments: 1) piglets provided with DPS in an optimal hygiene (OPTIMAL) farrowing accommodation (*n* = 22), 2) piglets provided with DPS in a sub-standard hygiene (SUB-STANDARD) farrowing accommodation (*n* = 23), 3) piglets provided with a liquid mixture of milk replacer and starter diet (LMR + S) in an optimal hygiene (OPTIMAL) farrowing accommodation (*n* = 21), and 4) piglets provided with a liquid mixture of milk replacer and starter diet (LMR + S) in a sub-standard hygiene (SUB-STANDARD) farrowing accommodation (*n* = 21). The experiment was a 2 × 2 factorial arrangement with the factors being pre-farrowing hygiene routine (SUB-STANDARD or OPTIMAL) and creep feeding regime (DPS or LMR + S). Where DPS was provided as creep feed or starter diet was used in the liquid diet (LMR + S), this diet was the nursery phase 1 diet (diet 3; Table 1) which was also fed for 1 week PW as outlined below. The creep feeding treatment groups were equally represented in each farrowing room. The litter size within each block was standardized by cross-fostering between 24 and 48 h after parturition. The final number of piglets per litter at 48 h postpartum was based on the rearing capacity and number of functional teats of each sow. No cross-fostering was performed between the SUB-STANDARD and OPTIMAL hygiene routines. The teeth of all piglets were clipped within 24 h of birth and on day 5 all of the piglets were injected with 1 mL of iron (Gleptosil, Ceva Santé Animale, Libourne, France) and the tails were docked. Male piglets remained entire and weaning was at 28 (±1.2) days after birth. At weaning, a subsample of 576 pigs (8.7 ± 1.5 kg) was selected and followed until target slaughter weight (~130 kg) to study the residual effects of the pre-weaning treatments in progeny. Within each treatment group, single-sex pen groups (12 male or female pigs of even weight per pen; *n* = 12 pens per treatment) were formed, and blocked on sex and weaning weight. Ten piglets per treatment were euthanized on day 4 PW to collect blood, intestinal tissue, and digesta samples. These 10 piglets per treatment were housed in four nursery/grower pens grouped by treatment, between weaning and day of euthanasia. All pigs were weighed on the day of euthanasia, and the feed intake (at a pen level) of each of these four nursery/grower pens between weaning and the day of euthanasia was recorded. The selection criteria for euthanized piglets and sampling methodology are outlined below.

**Table 1.** Composition of the experimental diets (on an as-is basis; kg/tonne unless otherwise stated)

Diet number Diet type	1 Gestation	2 Lactation	3^1^ Nursery phase 1	4 Nursery phase 2	5 Grower	6 Finisher
Ingredients						
Barley	759.7	259.7	50.0	68.4	292.7	589.7
Wheat	0.0	455.2	0.0	100.0	450.0	230.0
Maize	0.0	0.0	231.0	300.0	0.0	0.0
Soybean meal	76.2	179.8	143.5	186.9	150.2	146.1
Full-fat soybean meal	0.0	0.0	130.8	70.0	50.0	0.0
Whey permeate	0.0	0.0	200.0	150.0	0.0	0.0
Skim milk powder	0.0	0.0	125.0	50.0	0.0	0.0
Soya hulls	125.3	0.0	0.0	0.0	0.0	0.0
Soya oil	14.0	66.0	85.0	38.2	23.2	10.0
Premix^2^	1.5	1.5	3.0	3.0	3.0	1.3
L-lysine HCl	2.3	5.0	6.2	6.7	6.3	4.7
DL-methionine	0.4	1.5	3.6	3.2	2.1	1.0
L-threonine	1.0	2.7	3.6	3.4	2.7	2.0
L-tryptophan	0.0	0.8	1.4	1.3	0.6	0.3
L-valine	0.0	2.7	1.3	1.3	0.7	0.0
Limestone flour	8.5	11.5	7.0	7.5	10.0	11.0
Mono dicalcium phosphate	7.0	8.5	5.5	7.0	5.3	1.0
Salt	4.0	5.0	3.0	3.0	3.0	3.0
Phytase^3^	0.05	0.05	0.05	0.10	0.10	0.10
Chemical composition						
Dry matter^4^	883.0	893.0	906.0	899.0	876.0	884.0
Crude protein^4^	125.0	163.0	183.0	184.0	172.0	158.0
Ash^4^	42.0	48.0	50.0	50.0	38.0	40.0
Ether extract^4^	33.7	85.4	100.96	63.4	46.8	27.8
Crude fiber^4^	87.0	26.0	15.0	20.0	26.0	31.0
Lys^5^	7.8	11.5	16.2	15.0	12.8	10.8
Met^5^	2.4	3.9	7.0	6.1	4.6	3.6
Cys^5^	2.5	3.0	2.7	2.9	3.1	2.9
Thr^5^	5.6	8.3	10.9	10.0	8.5	7.5
Trp^5^	3.7	3.4	3.7	3.4	2.6	2.2
Net energy^5^, MJ/Kg	8.9	10.9	12.1	11.0	10.3	9.8
SID lys^5^	6.6	10.7	15.3	14.1	12.0	10.0

^1^Nursery phase 1 diet was the dry pelleted starter diet provided as creep feed and was the starter diet used to prepare the liquid mixture supplemented to suckling piglets.

^2^Premix provided per kilogram of complete diet 1 and 2: Cu from copper sulfate, 15 mg; Fe from ferrous sulfate monohydrate, 70 mg; Mn from manganese oxide, 62 mg; Zn from zinc oxide, 80 mg, I from potassium iodate, 0.6 mg; Se from sodium selenite, 0.2 mg; vitamin A as retinyl acetate, 3.44 mg; vitamin D3 as cholecalciferol, 25 mg; vitamin E as DL-alpha-tocopheryl acetate, 100 mg; vitamin K, 2 mg; vitamin B12, 15 μg; riboflavin, 5 mg; nicotinic acid, 12 mg; pantothenic acid, 10 mg; choline chloride, 500 mg; biotin, 200μg; folic acid, 5mg; vitamin B1, 2 mg; and vitamin B6, 3 mg.

Premix provided per kilogram of complete diet 3, 4, and 5: Cu from copper sulfate, 85 mg; Fe from ferrous sulfate monohydrate, 90 mg; Mn from manganese oxide, 47 mg; Zn from zinc oxide, 120 mg; I from potassium iodate, 0.6 mg; Se from sodium selenite, 0.3 mg; vitamin A as retinyl acetate, 2.1 mg; vitamin D3 as cholecalciferol, 25 μg; vitamin E as DL-alpha-tocopheryl acetate, 100 mg; vitamin K, 4 mg; vitamin B12, 15 μg; riboflavin, 2 mg; nicotinic acid, 12 mg; pantothenic acid, 10 mg; choline chloride, 250 mg; vitamin B1, 2 mg; and vitamin B6, 3 mg.

Premix provided per kilogram of complete diet 6: Cu from copper sulfate, 15 mg; Fe from ferrous sulfate monohydrate, 24 mg; Mn from manganese oxide, 31 mg; Zn from zinc oxide, 80 mg; I from potassium iodate, 0.3 mg; Se from sodium selenite, 0.2 mg; vitamin A as retinyl acetate, 0.7 mg; vitamin D3 as cholecalciferol, 12.5 μg; vitamin E as DL-alpha-tocopheryl acetate, 40 mg; vitamin K, 4 mg; vitamin B12, 15 μg; riboflavin, 2 mg; nicotinic acid, 12 mg; pantothenic acid, 10 mg; vitamin B1, 2 mg; and vitamin B6, 3 mg.

^3^Ronozyme HiPhos GT (Inform Nutrition, Whites Cross, Ireland) was included to provide 500 phytase units (FYT) per kg of diet.

^4^Analyzed chemical composition.

^5^Calculated chemical composition from book values for ingredients (Sauvant et al., 2004).

### Cleaning protocols used in the farrowing rooms

The SUB-STANDARD and OPTIMAL hygiene routines were obtained by implementing a basic cleaning protocol or an optimal cleaning protocol, respectively, prior to entry of the sows to the farrowing rooms, as described previously (Halpin et al., 2024) but with a slight modification to the optimal cleaning protocol. The basic cleaning protocol involved washing the pens with cold water using a power washer (Triace 13 hp, Triace, Fermoy, Ireland). No detergent or disinfectant was used and ~18 h of drying time was allowed but without any supplementary heat. The optimal cleaning protocol involved a pre-soaking stage, during which the floor and wall surfaces were rinsed down with water and allowed to soak for 18 h. The following day, a 1% solution of a carboxylic acid-based detergent (Blast Off; Biolink Ltd, Sutton Fields, UK) was applied to the floor and wall surfaces of each pen in the room. After a contact time of 20 min, the pen surfaces were thoroughly washed with cold water using a power washer. The pens were allowed to dry for 3 days followed by application of a 3% solution of a chlorocresol-based disinfectant (Interkokask; Interhygiene GmbH, Cuxhaven, Germany). After disinfection, pens were allowed to dry for 3 more days. During the drying time, the temperature of the farrowing room was maintained at ~24 °C and the temperature of the heat pads at 38 to 40 °C. As in the Halpin et al. (2024) study, on the day of transfer to the farrowing room, the sows in the OPTIMAL treatment were washed gently with water using a power washer and then thoroughly disinfected using 0.5% Virkon S (Lanxess, Cologne, Germany) using a knapsack sprayer (Pressure Sprayer; Draper Tools, Chandler’s Ford, UK). The sows in the SUB-STANDARD treatment were not washed or disinfected. The same hygiene protocol (SUB-STANDARD or OPTIMAL) was applied to all pens within a farrowing room and the pen hygiene conditions were verified by microbiological analysis of pen swabs as outlined below. The allocation of the hygiene protocols to the farrowing room was reversed in the subsequent farrowing group.

### Experimental diets

All diets were formulated to meet or exceed recommendations of the National Research Council (NRC, 2012). Representative samples were collected from all diets and were analyzed for DM (oven drying), ash (furnace drying and gravimetry), crude protein (Dumas method), total fat (Wiebul acid hydrolysis), and crude fiber (Ankom 200 fiber analyzer, Macedon, USA) by Sciantec Analytical services Ltd, Selby, United Kingdom according to the European Union Commission Regulation No. 152/2009 of 27 January 2009. The ingredient composition and nutrient content of the experimental diets are presented in Table 1. A gestation diet (diet 1; Table 1) in meal form was fed to sows at a feed allowance of 2.65 kg/day (35 MJ/day of digestible energy) between day 0 of gestation and parturition. During gestation, feed was provided from two electronic sow feeders (Schauer feeding system, Prambachkirchen, Austria). On day 110 of gestation, sows were moved to the farrowing accommodation and were fed using a computerized feed delivery system (DryExact Pro, Big Dutchman, Vechta, Germany). Post-farrowing, sows were fed a lactation diet (diet 2; Table 1) in meal form twice daily until day 6 of lactation and three times daily from day 7 until weaning at 28 days. The sow lactation feeding curve started at 58 MJ/day on day 0 of lactation and gradually increased to 104, 121, 129, and 133 MJ/day of digestible energy on days 7, 14, 21, and 26 of lactation, respectively. The feeding curves for individual sows were adjusted regularly during lactation, as required, in order to prevent feed wastage and to ensure feed allowance approximated *ad-libitum* feeding. Nursery phase 1/DPS (diet 3; Table 1) was provided as 3-mm diameter pellets (DPS) and was also used to prepare the liquid mixture of milk replacer and starter diet (LMR + S). The DPS-fed piglets were provided with creep feed from day 11 after birth until weaning in a circular feeder (Easy Pan, Rotecna, Lleida, Spain) located at the bottom corner of the heat pad to one side of the sow’s crate. The disappearance of DPS creep feed for each litter was determined weekly and every effort was made to minimize feed wastage by manually providing DPS up to four times a day (~50 to 100 g per pen at each feeding) and the quantity of feed provided was increased as feed disappearance increased over time. Day 11 after farrowing was selected to start creep feeding DPS to conform with standard farm practices where dry creep feeding generally commences after day 10 post-farrowing, since manual creep feeding is very labor intensive and creep feed intake prior to day 10 is extremely low. However, as automated liquid feed delivery systems allow earlier commencement of liquid feeding, we opted to commence automated liquid creep feeding from day 4 after farrowing by which time piglets would have consumed sufficient quantities of colostrum. The objective here was to compare a standard manual dry creep feeding regime with a state-of-the-art automated liquid creep feeding regime. The LMR + S feed was provided in a semicircular feed trough located at the front of the pen at one side of the sow’s head as a liquid mixture (~15.4% DM) of milk replacer (Opticare milk; Swinco B.V, Venray, The Netherlands) and an increasing proportion of starter diet between day 4 and weaning. The proportion of milk replacer in the LMR + S was 100%, 75%, 50%, 25%, and 0% during the period from day 4 to 9, day 10 to 14, day 15 to 17, day 18 to 21, and day 22 to 28, respectively. The exact proportion of milk replacer powder and starter diet mixed with water on a fresh weight basis for each period during lactation is outlined in Supplementary Table S1. The milk replacer powder used contained the following, in descending order of inclusion: sweet whey powder, vegetable oils, porcine dried plasma powder, whey powder, digestible starch, dextrose, hyper-immunized egg powder, soya protein concentrate, hydrolyzed wheat gluten, premix of amino acids, vitamins, and trace minerals. The milk replacer powder contained 11.9 MJ net energy per kg, 21.5% crude protein, 9% fat, 0.1% crude fiber, 6.5% crude ash, 1.8% lysine, 0.46% methionine, 0.7% calcium, 0.55% phosphorus, and 0.7% sodium.

After weaning, pigs were fed a series of dry pelleted diets according to their growth stage. Nursery phase 1 (diet 3; Table 1) was provided from the day of weaning to day 7 PW, a nursery phase 2 diet (diet 4; Table 1) from day 7 to day 21 PW, a grower diet (diet 5; Table 1) from day 21 to day 47 PW and a finisher diet (diet 6; Table 1) from day 47 PW to slaughter (~day 131 PW). The four pens of pigs that were euthanized were also fed with the nursery phase 1 diet, between weaning and the day of euthanasia (day 0 to 4 PW).

### Pre-weaning liquid feeding system

The LMR + S was provided by an automated delivery system (Babyfeed; Schauer Agrotronic GmbH, Prambachkirchen, Austria), where feed was delivered to semicircular feed troughs either left or right of the sow’s head. Two fresh mixes of liquid feed were prepared each day (at 9:00 h and 17:00 h) by mixing the ingredients with warm water at 55 °C. Each liquid feeding cycle occurred between 9:30 h and 4:00 h. A sensor was installed above the liquid feeder troughs which checked the level of liquid feed present in the trough every 24 min. Fresh LMR + S was delivered to the trough, whenever the liquid level was detected to be below the sensor level. Hence, each pen was potentially supplied with LMR + S up to 50 times each day, ensuring *ad-libitum* feeding. Every morning at ~6:00 h, the system was cleaned in closed circuit, including the mixing tanks and the pipelines, with a 1% acid solution (Deosan Acidbrite AG313, Diversey Europe Operations BV, Utrecht, The Netherlands). In addition, the system was cleaned once a week with a 0.5% solution of an alkaline detergent (AvalKsan, Gold Standard CF, Carbon Group, Ringaskiddy, Ireland), in order to remove limescale from the circuit. After cleaning the system every morning (~8.30 h), the feed troughs were also cleaned with air pressure and were rinsed with acid solution every day and alkaline solution once a week as outlined above. Before the cleaning of the troughs, any leftover unconsumed feed was quantified in each trough.

### Housing

Sows were placed in farrowing crates (BigDutchman) in individual farrowing pens (dimensions: 2.5 m × 1.8 m) from day 107 of gestation until weaning. Water on an *ad-libitum* basis was provided to sows from a single-bite drinker in the feed trough and to suckling piglets from a bowl drinker in the farrowing pens. The sow lying area of the farrowing pen consisted of cast iron slats, while the remaining farrowing pen consisted of a plastic slatted floor with a water-heated floor pad for the piglets (BigDutchman). Temperature was automatically controlled in all of the rooms and ventilation was provided by punched ceiling ventilation with air exhausted by a variable speed fan linked to a thermostat and controlled automatically with a controller outside each room (Big Dutchman 135). The farrowing room temperature was maintained at ~24 °C during farrowing and was gradually reduced to 22.5 °C by day 7 of lactation. The temperature of the heat pads was maintained at 35 to 38 °C during lactation. Environmental enrichment in the farrowing room consisted of a star-shaped plastic toy (Easyfix Luna 142, Easyfix, Galway, Ireland) attached to a metal chain mounted on the side of the pen for the piglets and a jute bag tied to the front of the crate for the sow.

The nursery/grower pens (dimensions: 2.5 m × 2 m) had fully slatted plastic floors, a shelf-type single-space (33 cm) wet-dry feeder (BA19100, Verba, Verbakel, Netherlands) with inset nipple drinker and an additional bowl drinker (SS Drinker, Rotecna). In the nursery/grower room, temperature was maintained at 28 °C in the first week after weaning and was reduced by 2 °C each week to 22 °C at the end of the 4th week PW. Enrichment in nursery/grower rooms consisted of two star-shaped plastic toys (Easyfix Luna 142) and a chain mounted on the side of the pens. At day 47 PW, pen groups were moved to finisher pens (dimensions: 2.4 m × 4.2 m) with fully slatted concrete floors, a shelf-type single-space (33 cm) wet-dry feeder (MA19100, Verba) with inset nipple drinker and an additional bowl drinker (SS Drinker, Rotecna). Temperature in the finisher rooms was maintained at 20 to 22 °C. Enrichment in finisher rooms consisted of a wooden (larch) post fixed to the wall of the pens. For all of the rooms, artificial lighting was provided by tubular LED fixtures daily from 08:00 h to 16:30 h.

### Data recording, sampling, and laboratory analysis

#### Microbiological analysis of swab samples from farrowing pens

A total of 32 farrowing pens (16 SUB-STANDARD and 16 OPTIMAL) were swabbed 1 day prior to entry of the sows to the farrowing pens. Two samples were collected from each farrowing pen which included floor swabs from the piglet lying area (2 × 100 cm^2^ swabs pooled) and floor swabs from the area behind the sow (3 × 100 cm^2^ swabs pooled). All swabbing was performed using sterile sponges pre-hydrated with 10-mL neutralizing buffer (3M, Bracknell, United Kingdom) and a 100 cm^2^ metal grid. All swabs were collected aseptically by sterilizing the grid with 70% ethanol and changing gloves between each sample. All sponge swabs were placed back into their sterile stomacher bags and sealed and transported to the laboratory on ice. Pooled sponge swabs from each area were suspended in 40 mL of maximum recovery diluent (MRD; Oxoid, Basingstoke, United Kingdom) and homogenized in a stomacher (Stomacher 400 Circulator; Seward Limited, Worthing, United Kingdom) at 260 rpm for 2 min. After homogenization, 1 mL of the sample was added to 9 mL of MRD and from that, 10-fold serial dilution was performed in MRD. Relevant dilutions were plated in duplicate as follows: 1) pour-plated on violet red bile dextrose agar (Merck, Darmstadt, Germany), overlaid, and incubated at 37 °C for 24 h for *Enterobacteriaceae*; and 2) spread-plated on Petrifilm aerobic count plates (3M Health Care, Minneapolis, USA) and incubated at 37 °C for 48 h for total bacterial counts. Colony-forming units (CFU) were counted and duplicate counts were averaged and presented as log_10_ CFU/cm^2^. The CFU/cm^2^ was calculated as: [(Average CFU/plate) × (volume of original suspension (40 mL)] / (Total swabbed surface area × dilution factor). The limit of detection calculated for the floor area behind the sow was 0.4 log_10_ CFU/cm^2^ and for the piglet lying area was 0.6 log_10_ CFU/cm^2^.

#### Sow bodyweight and back fat depth

Sow bodyweight and back fat depth were recorded on day 110 of gestation and at weaning as outlined by Arnaud et al. (2024).

#### Mortality, medication usage, and prevalence of diarrhea

All piglet deaths and antibiotic and anti-inflammatory usage, in terms of milliliters of medication used per pig and number of clinical cases of disease per litter (number of pigs that were treated on one or more occasion), were recorded. The only antibiotic used was Unicillin (Procaine Benzylpenicillin, 300 mg/mL injection, Univet, Cootehill, Cavan, Ireland) and the only anti-inflammatory used was Loxicom 5 mg/mL injection (Norbrook, Monaghan, Ireland). The antibiotic and anti-inflammatory usage values were averaged by dividing the total volume used to treat the piglets in each pen by the number of piglets in each pen. The percentage mortality from day 4 to weaning for each litter was calculated as the percentage of deaths based on litter size per sow at day 4 after birth. The percentage mortality from weaning to slaughter for each PW pen group was calculated as follows: total number of deaths per pen group divided by 12 (number of pigs per pen at weaning). Visual scoring of fecal consistency at a pen level was performed on days 11, 18, and 28 after birth and on days 4, 7, 9, and 14 PW. A four-grade scoring system (Casey et al., 2007) was used as follows: 0 for dry pelleted feces; 1 for soft feces with shape; 2 for mild diarrhea (very soft without shape or viscous liquid feces); and 3 for severe diarrhea (watery or with blood). The average score from five pigs per litter/pen was determined to provide the average score for each litter/pen. The diarrhea prevalence at each time point was determined by considering a fecal score of 2 or greater as indicative of diarrhea for each litter/pen. The overall diarrhea prevalence was then calculated for the pre-weaning period between days 11 and 28 and the early PW period between days 4 and 14 PW.

#### Pre-weaning growth performance

Suckling piglets were weighed individually at birth, on day 4, and on day 28 after birth using an electronic weighing scale (Defender 3000 XtremeW, O’Donovan Engineering, Coachford, Ireland). Creep feed disappearance for DPS was recorded by performing feed weighbacks on days 18 and 28 after birth. Creep feed disappearance for LMR + S was recorded daily by the liquid feeding system between days 4 and 28. The total DM intake (TDMI) during the entire pre-weaning period per pig per litter was calculated from feed weigh backs and feed downloads from the liquid feeding system on a DM basis. Using the TDMI, total net energy intake and total lysine intake from creep feed were calculated. The average daily feed intake (ADFI) from day 4 to 28 and from day 11 to 28 was calculated on a DM basis. Average daily gain (ADG) of piglets in each litter was calculated using average piglet bodyweight of each litter on each weighing day. The coefficient of variation (CV) of within-litter piglet bodyweight was calculated to determine litter uniformity.

#### Live observation of feeding behavior

Live observations of feeder-directed behavior of individual piglets were recorded on days 6, 13, 20, and 27 after birth for the first three farrowing groups. On each observation day, six 1-h sessions of live observations were conducted between 9:00 h and 16:00 h, using 3-min instantaneous scan sampling. During each 1-h session, each pen was scanned every 3 min, leading to 21 scans of a pen per session and 126 scans per day. To facilitate individual identification, piglets were individually marked with numbers on their backs using black hair dye (Pro Colour Plus 0.1, Wolverhampton, United Kingdom) prior to the day of observations. A simple ethogram was used for recording feeding behavior of individual piglets. At each scan, piglets in each litter actively engaging in feeder-directed activity (snout positioned inside the feeder trough) were counted. Sleeping or inactive piglets with their snouts in the feeder trough were excluded. Two observers were trained prior to the day of observations by practicing scanning on the same 14 litters for one session. These two observers performed the scan sampling. One observer scan sampled litters in one farrowing room and the other observer scan sampled litters in the other farrowing room. Observers switched farrowing rooms prior to each of the six daily sessions. The percentage of piglets observed to engage in feeder-directed activity in each litter per observation day was calculated by dividing the number of piglets observed engaging in feeder-directed activity at least once during the day by the litter size and multiplying by 100.

#### Post-weaning growth performance and carcass data

Pen groups were weighed on day 0 (weaning) and on the day of slaughter (~day 131 PW) using an electronic weighing scale (EziWeigh 7i, O’Donovan Engineering). Pigs were fasted for ~12 h prior to recording bodyweight on the day of slaughter. Feed disappearance in each pen was recorded from weaning until slaughter day. These data were used to calculate the ADFI, ADG, and gain to feed ratio (G:F) for each pen. Entire pen groups were slaughtered over a 3-week period (day 126 or 133 or 140 PW). As per routine farm practice, the heaviest pens of pigs were sold first based on treatment-blinded visual assessment by farm staff. The weaning to slaughter duration (days) was calculated for each pen. On the day of slaughter, pigs were transported 95 km to the abattoir (Dawn Pork & Bacon, Grannagh, Ireland) and they were slaughtered by CO_2_ stunning followed by exsanguination. Cold carcass weight, kill-out percentage, muscle and back fat depth, and lean meat percentage were calculated as described previously by Vasa et al. (2024).

#### Fecal sampling

Fecal samples were collected from 15 focal pigs per treatment of median birth weight for their litter 2 days before weaning (Weaning), day 21 PW and prior to slaughter at day 114 PW. Whenever possible, freshly voided fecal samples were collected and if this was not possible, then samples were obtained by digital rectal stimulation. All samples were collected into sterile 50-mL plastic tubes and kept on ice. Subsamples were then transferred into sterile 2-mL tubes, immediately snap-frozen in liquid nitrogen (within 5 min of collection) and stored at −80 °C until DNA extraction. Out of the 15 focal pigs/treatment, fecal samples from ~8 focal pigs/treatment (balanced sex ratio) which were observed to be consuming DPS or LMR + S creep feed and remained healthy until sale were selected for DNA extraction. The ~8 focal pigs/treatment represented seven to eight litters per treatment.

#### Euthanasia and tissue sampling

On day 4 PW, 10 female pigs/treatment (median weaning weight of their litter) which were observed to be consuming DPS or LMR + S creep feed during lactation were euthanized to collect blood, intestinal tissue, and digesta samples. The 10 pigs/treatment represented four to five litters per treatment. Pigs were euthanized using captive bolt followed by immediate exsanguination, during which ~9 mL of whole blood was collected into vacutainer tubes containing ethylene diamine tetra acetic acid (Becton-Dickson Ltd, Franklin Lakes, USA). The intestinal tract was removed and two sets of whole tissue samples (~2 cm) were excised from the duodenum (15 cm distal to the pyloric junction), jejunum (1.5 m distal to the pyloric junction), and ileum (15 cm proximal to the ileocecal junction). One set of whole tissue samples from each region was rinsed in cold phosphate buffered saline and placed in NOTOXhisto, an alcohol/aldehyde fixative (Scientific Device Laboratory, Des Plaines, IL, USA), and was placed on a shaker for 48 h for histological analysis. The other set of tissue samples from each region was longitudinally opened and, using a glass slide, the mucosal layer was scraped into 2-mL tubes, snap-frozen in liquid nitrogen, and stored at −80 °C for enzyme activity assays. Digesta from jejunum and ileum was collected into sterile 2-mL tubes, immediately snap-frozen in liquid nitrogen, and stored at −80 °C until DNA was extracted.

#### Hematological analysis of blood samples

Hematological analysis of the blood samples collected from euthanized piglets was performed using a Mythic 5 vet pro analyzer (Cormay Diagnostics, Warsaw, Poland) within 5 to 7 h after collection. The following parameters were measured: leukocyte count, lymphocyte count and percentage, monocyte count and percentage, neutrophil count and percentage, eosinophil count and percentage, basophil count and percentage, erythrocyte count, hemoglobin (g/dL), mean corpuscular volume (fL), mean corpuscular hemoglobin (pg/cell), mean corpuscular hemoglobin (g/dL), and platelet count.

#### Small intestinal histology

The whole tissue samples placed in NOTOXhisto were sent to Nationwide Laboratories, Devon, UK for histological slide preparation and hematoxylin and eosin staining. Gross morphological parameters of intestinal structure were studied using light microscopy (Olympus Plus BX51TF, Olympus Corporation, Tokyo, Japan). Ten intact villi and crypts were identified on five fields of view from each intestinal sample and the villus height (VH), villus width (VW), and crypt depth (CD) were measured using ProgRes CapturePro software (version 2.10.0.0, JENOPTIK optical systems GmbH, Jena, Germany). The average VH, VW, CD, and ratio of VH:CD was calculated for each sample.

#### Enzyme activity analysis

Mucosal scrapings were weighed and brush-border membrane enzymes were extracted by homogenizing with 1% Triton-X buffer. The tissue-buffer homogenates were centrifuged at 2000G for 10 min at 4 °C and the supernatant was used to determine the enzyme activities of three disaccharidase enzymes (sucrase, maltase, and lactase), as described in detail previously by Vasa et al. (2024). In brief, the specific substrates (lactose, maltose, and sucrose) were added to the supernatant from the tissue sample and incubated for 30 min at 37 °C. After incubation, 250 μL of peroxidase–glucose oxidase color solution was added and the samples were spectrophotometrically analyzed at 450 nm and 37 °C using a plate reader (SpectraMax ABS, VWR International, Radnor, PA, USA). All enzyme activities were calculated using the slope and intercept of a glucose standard curve and expressed as enzyme units per gram of tissue (U/g). All of the reagents and substrates used were supplied by Merck Life Sciences Limited (Arklow, Ireland).

#### Microbiota analysis of fecal and digesta samples

Total DNA was extracted from 0.4 g of digesta and fecal samples using the QIAamp PowerFecal Pro DNA kit (Qiagen, Crawley, United Kingdom), according to the manufacturer’s instructions. The concentration of extracted DNA was measured using a Simplinano Spectrophotometer (Biochrom Ltd., Cambridge, United Kingdom). The purity of DNA was assessed using the 260/280 and 260/230 nm ratios of absorbance measured by the same spectrophotometer. The samples were then diluted to a standardized concentration of 20 ng/µL in nuclease-free water (Thermo Fisher Scientific, Waltham, MA, USA) and stored at −20 °C until library preparation and sequencing. Three jejunal digesta samples (two from DPS_OPTIMAL group and one from LMR + S _OPTIMAL group) were not sequenced as their DNA concentration was too low.

The PCR amplification, library preparation, and sequencing were performed by the VIB Nucleomics Core (Leuven, Belgium). The PCR amplification of bacterial full-length 16S rRNA genes (V1–V9 region) was performed using a KAPA HiFi HotStart ReadyMix PCR kit (Roche Sequencing and Life Sciences, Indianapolis, IN, USA) according to the Pacific Biosciences of California (PacBio) Procedure & Checklist (Part Number 101-599-700 Version 05). The F27 (AGRGTTYGATYMTGGCTCAG) and R1492 (RGYTACCTTGTTACGACTT) universal primer set was used and both the forward and reverse primers were tailed with sample-specific PacBio barcode sequences for multiplexed sequencing. The amplicons were then pooled in equimolar concentrations and the multiplexed amplicon libraries were constructed using the SMRTbell prep kit 3.0 (PacBio, Menlo Park, USA) with SMRTbell barcoded adapters (PacBio), following the PacBio Procedure & Checklist (Part Number 102-359-000 Version 02). The libraries were loaded onto a PacBio Sequel IIe device using the Sequel II binding kit 3.2 (PacBio) and the V1-V9 region of the 16S rRNA gene was sequenced.

Pacific Bioscience data were de-multiplexed and circular consensus sequences were called using the SMRT-Link analysis software (v9). Resulting FASTQ forma files (available at: https://www.ebi.ac.uk/ena/browser/view/PRJEB78943) were processed using the DADA2 package (version 1.22.0) in R (version 4.3). The “removePrimers” function was used to identify and trim both forward (27F) and reverse (1492R) primers and remaining FASTQ sequences were reoriented in a consistent direction. The “filterAndTrim” function was used to generate a high-quality subset of reads based on expected read length and sequence quality trimming. The DADA2 PacBio error model was used to estimate sequence errors and samples were de-replicated and sample composition was estimated using full pooling methods. Chimeric sequences were removed using the “removeBimeraDenovo” function and taxonomic classification of remaining amplicon sequence variants (ASVs) was subsequently performed using the “assignTaxonomy” function from DADA2 against the Genome Taxonomy Database using the recommended minimum bootstrap cutoff of 80. Species/strain/serotype level identification was performed by searching the exact sequence of relevant genera in the Basic Local Alignment Search Tool (BLAST) nucleotide database of the U.S. National Center for Biotechnology Information (available at: https://blast.ncbi.nlm.nih.gov/Blast.cgi). DADA2 output files were converted into a Phyloseq object for downstream analysis. Low abundance ASVs (< 10 reads) were manually filtered and ASVs were retained if their relative abundance was greater than 0.005%. Three more jejunal digesta samples (one from DPS_ SUB-STANDARD group and two from LMR + S _OPTIMAL group) were removed during the ASV filtration step due to too few reads.

### Statistical analysis

Statistical analyses were conducted using SAS version 9.4 (SAS Institute Inc. Cary, NC, USA). All data were tested for normality using the univariate procedure and the normal distribution and residuals plots were inspected. Growth performance indicators in the pre-weaning and PW period which were recorded at multiple time points were analyzed as repeated measures using the PROCMIXED procedure for a 2 × 2 factorial arrangement. The model included creep feeding regime (DPS, LMR + S), hygiene (SUB-STANDARD, OPTIMAL), and their associated interactions as fixed effects. Block was included as a random effect and piglet birth weight (for pre-weaning parameters) and weaning weight (for PW parameters) were included as co-variates, when significant in the model. Day after birth (for pre-weaning parameters) or day after weaning (for PW parameters) was included in the above models as a repeated variable. The percentage of piglets within each pen observed to engage in feeder-directed behavior was also recorded at multiple time points and therefore was analyzed as repeated measures using the same procedure as described above. The ADG and ADFI for the total pre-weaning period, ADFI from day 11 to 28, TDMI, bacterial counts from pen swabs (log_10_ CFU/cm^2^), antibiotic usage, anti-inflammatory usage, and number of clinical cases of disease were analyzed using the same model as described above but without time as a repeated variable. For sow bodyweight and back fat depth post-farrowing and at weaning, the same procedures as described in the model above were used but with sow bodyweight and back fat depth at day 110 of gestation included as co-variates, when significant in the model. For all of the above-described parameters, the litter/sow was the experimental unit prior to weaning and the pen group was the experimental unit PW. The histological parameters, brush-border enzyme activities, hematology parameters, and ADG of euthanized pigs between day 0 and 4 PW were analyzed using the fixed effects as described in the model above, but with litter/sow as a random effect and weaning weight included as a covariate when significant in the model. The individual piglet was the experimental unit. In all of the mixed models, appropriate co-variance structure as indicated by the model fit statistics was applied and Tukey–Kramer adjustment was applied for multiple comparison of means. The prevalence of diarrhea in the pre-weaning period between days 11 and 28 and the early PW period between days 4 and 14 PW was analyzed using PROC GLIMMIX with binomial distribution. Pre-weaning mortality percentage per litter and weaning to slaughter duration were analyzed using PROC GLIMMIX with multinomial distribution.

For microbiome data, alpha (observed, Shannon, and inverse-Simpson) indices and beta (Bray–Curtis distance) diversity metrics were estimated using the microeco and phyloseq packages in R (version 4.3). Comparisons of various diversity metrics between treatment groups were carried out using the Adonis function in the Vegan R package, Wilcox rank sum tests in the microeco R package, and lm function from the stats library. Differential abundance estimates between treatment groups were carried out using both the LinDa and DeSeq2 R packages. Visualization of results using PCoA and box plots was carried out using the R ggplot2 package. Significant differences were considered when *P* ≤ 0.05 with 0.05 < *P* ≤ 0.1 considered as tendencies.

## Results

### Microbiological analysis of swab samples from farrowing pens

*Enterobacteriaceae* and total bacterial counts on both of the sampled pen floor areas just before entry of the sows to the farrowing pens are presented in Supplementary Figure S1. The *Enterobacteriaceae* count from the piglet lying area was 1.8 log_10_ CFU/cm^2^ lower in the OPTIMAL compared to the SUB-STANDARD hygiene pens (*P* ≤ 0.05) and 3.1 log_10_ CFU/cm^2^ lower from the floor area behind the sow (*P *≤ 0.05). The total bacterial count from the piglet lying area was 2.0 log_10_ CFU/cm^2^ lower in the OPTIMAL compared to the SUB-STANDARD hygiene pens (*P* ≤ 0.05) and 2.8 log_10_ CFU/cm^2^ lower from the floor area behind the sow (*P* ≤ 0.05).

### Sow bodyweight and back fat depth

The effect of creep feeding and hygiene and their associated interactions on sow bodyweight and back fat depth are presented in Supplementary Table S2. No significant effects were observed.

### Mortality, medication usage, and prevalence of diarrhea

The effect of creep feeding and hygiene and their associated interactions on medication usage, clinical cases of disease, and prevalence of diarrhea in suckling piglets is presented in Table 2. There was no creep feeding × hygiene interaction or main effect of creep feeding for antibiotic or anti-inflammatory usage per pig or number of clinical cases of disease per litter during the pre-weaning period. Piglets born into the OPTIMAL hygiene tended to have lower antibiotic and anti-inflammatory usage compared to those born into the SUB-STANDARD hygiene during the pre-weaning period (*P* = 0.08). The number of clinical cases of disease per litter was lower in the OPTIMAL compared to the SUB-STANDARD hygiene during the pre-weaning period (*P* ≤ 0.05). There was a tendency for a creep feeding × hygiene interaction for prevalence of diarrhea during the pre-weaning period (*P* = 0.08). Litters fed with LMR + S in the OPTIMAL hygiene tended to have a lower prevalence of diarrhea compared to the LMR + S-fed piglets in the SUB-STANDARD hygiene and the DPS-fed piglets in both the OPTIMAL and SUB-STANDARD hygiene. Litters in the OPTIMAL hygiene had a lower prevalence of diarrhea compared to those in the SUB-STANDARD hygiene during the pre-weaning period (*P* ≤ 0.05). However, there was no effect of creep feeding regime on prevalence of diarrhea during the pre-weaning period. There was no creep feeding × hygiene interaction for mortality from day 4 to weaning and no main effect of hygiene (Table 3). However, there was a main effect of creep feeding, where LMR + S-fed litters had lower mortality than DPS-fed litters between day 4 and weaning (*P* ≤ 0.05). Causes of death during the pre-weaning period included starvation, crushing, leg injuries, and meningitis.

**Table 2.** Effect of creep feeding regime (DPS or LMR + S) and pre-farrowing hygiene routine (SUB-STANDARD or OPTIMAL) on medication usage in suckling piglets [Least square means ± pooled standard errors of the mean (SEM)]

Main effects of hygiene and creep feeding^1^	OPTIMAL	SUB-STANDARD	SEM for hygiene	DPS	LMR + S	SEM for creep feed	*P*-value		
							Hygiene	Creep feed	Hygiene × Creep feed
Number of sows/litters	43	44		45	42				
Antibiotic usage^2^, mL/pig/litter	0.17	0.28	0.04	0.27	0.18	0.04	0.08	0.13	0.55
Anti-inflammatory usage^2^, mL/pig/litter	0.03	0.06	0.01	0.05	0.04	0.01	0.08	0.13	0.55
Number of clinical cases^3^, number/litter	1.53	2.67	0.37	2.27	1.93	0.37	0.03	0.49	0.67
Diarrhea prevalence from day 11 to day 26 after birth^4^, %	16.7	31.9	3.7	27.2	20.0	3.8	0.01	0.19	0.08

^1^DPS = Suckling piglets provided with dry pelleted starter diet from day 11 to 28 of age; LMR + S = Suckling piglets provided with a liquid mixture of milk replacer and starter diet from day 4 to 28 of age; OPTIMAL = Suckling piglets born in farrowing accommodation cleaned with an optimal hygiene routine; SUB-STANDARD = Suckling piglets born in farrowing accommodation cleaned with a sub-standard hygiene routine.

^2^The only antibiotic used was Unicillin (Procaine Benzylpenicillin, 300 mg/mL injection, Univet, Cootehill, Cavan, Ireland) and the only anti-inflammatory used was Loxicom 5 mg/mL injection (Norbrook, Monaghan, Ireland). The values were averaged by dividing the total volume used per litter by the number of piglets in each litter.

^3^The number of pigs in each litter that were treated on one or more occasions.

^4^Visual scoring of fecal consistency at pen level was recorded on days 11, 18, and 28 after birth using a four-grade scoring system (Casey et al., 2007) as follows: 0 for dry pelleted feces; 1 for soft feces with shape; 2 for mild diarrhea (very soft without shape or viscous liquid feces); and 3 for severe diarrhea (watery or with blood). Prevalence of diarrhea (%) was calculated for each treatment by dividing the number of fecal score of 2 or 3 by the total number given from day 11 to day 26 after birth and multiplying by 100.

**Table 3.** Effect of creep feeding (DPS or LMR + S) and pre-farrowing hygiene routine (SUB-STANDARD or OPTIMAL) on feed intake and growth performance of suckling piglets [Least square means ± pooled standard errors of the mean (SEM)]

Creep feeding^1^	DPS		LMR + S			*P*-value		
Hygiene^1^	OPTIMAL	SUB-STANDARD	OPTIMAL	SUB-STANDARD	SEM	Hygiene	Creep feed	Hygiene × Creep feed
Number of sows/litters	22	23	21	21				
Bodyweight, kg								
Day 4	2.12	2.17	2.29	2.19	0.09	0.78	0.32	0.62
Day 28 (weaning)	8.31^ab^	8.22^a^	8.93^c^	8.67^bc^	0.18	0.35	<0.01	0.03
Overall					0.09	0.02	0.03	0.50
ADFI^1^ from day 4 to 28 (DM^1^ basis), g/pig/day	16^a^	15^a^	26^b^	13^a^	2.09	0.01	0.08	0.01
ADFI^1^ from day 11 to 28 (DM^1^ basis), g/pig/day	23^a^	21^a^	31^b^	16^a^	2.72	<0.01	0.44	0.01
TDMI^1^ (DM^1^ basis), g/pig	374^a^	343^a^	576^b^	310^a^	46.18	0.01	0.09	0.02
Total net energy intake from creep feed^2^, MJ/pig	4.3^a^	4.3^a^	7.0 ^b^	3.7^a^	0.59	0.01	0.07	0.01
Total lysine intake from creep feed^2^, g/pig	5.8^a^	5.8^a^	9.9^b^	5.2^a^	0.82	0.01	0.01	0.04
ADG^1^ day 4 to 28, g/pig/day	274	269	295	284	7.9	0.21	0.01	0.65
Coefficient of variation of individual piglet bodyweight within litter, %								
Day 4	21.2	21.0	18.6	19.8	1.36	0.71	0.16	0.49
Day 28 (weaning)	21.4^a^	18.8^ab^	18.1^ab^	15.9^b^	1.14	0.44	0.13	0.01
Overall					1.07	0.39	0.02	0.68
Mortality between day 4 and weaning, %	5.0	3.2	2.6	2.3	0.51	0.64	0.05	0.41

^1^DPS = Suckling piglets provided with dry pelleted starter diet from day 11 to 28 of age; LMR + S = Suckling piglets provided with a liquid mixture of milk replacer and starter diet from day 4 to 28 of age; OPTIMAL = Suckling piglets born in farrowing accommodation cleaned with an optimal hygiene routine; SUB-STANDARD = Suckling piglets born in farrowing accommodation cleaned with a sub-standard hygiene routine; ADG = Average daily gain; ADFI = Average daily feed intake; TDMI = Total dry matter intake; DM = dry matter.

^2^Calculated chemical composition values.

^a–c^Values within a row that do not share a common superscript differ significantly at *P* ≤ 0.05.

There was no effect of hygiene or creep feeding or their associated interactions on percentage mortality/removals from weaning to slaughter (data not shown). The mortality/removals percentage between weaning and slaughter was 9.7%, 4.2%, 4.9%, and 8.3% (SEM = 2.9) for DPS-fed piglets in OPTIMAL hygiene, DPS-fed piglets in SUB-STANDARD hygiene, LMR + S-fed piglets in OPTIMAL hygiene and LMR + S-fed piglets in SUB-STANDARD hygiene, respectively. Deaths and removals during the PW period were due to tail-biting, lameness/leg injury, and meningitis. The effect of treatment on medication usage, clinical cases of disease, and prevalence of diarrhea in weaned pigs is presented in Supplementary Table S3. There was a tendency for a creep feeding × hygiene interaction for antibiotic usage (*P* = 0.08) and number of clinical cases of disease (*P* = 0.09) from weaning to slaughter. Pigs fed with DPS from the OPTIMAL farrowing pens tended to have higher antibiotic usage than the other three treatment groups and tended to have a higher number of clinical cases of disease than pigs fed with LMR + S originating from the OPTIMAL farrowing pens. There was no main effect of hygiene or creep feeding on medication usage or clinical cases of disease during the PW period. There was no creep feeding × hygiene interaction for prevalence of diarrhea from day 4 to 14 PW and no main effect of creep feeding. However, there was a main effect of hygiene, whereby pigs from the OPTIMAL hygiene farrowing pens had a lower prevalence of diarrhea from day 4 to 14 PW compared to those from the SUB-STANDARD hygiene farrowing pens (*P* ≤ 0.05).

### Feed intake and growth performance of suckling piglets

The effects of creep feeding regime and hygiene and their associated interactions on pre-weaning piglet feed intake and growth are presented in Table 3. There was a creep feeding × hygiene interaction for ADFI from day 4 to 28, ADFI from day 11 to 28, TDMI, total energy intake, and total lysine intake (*P* ≤ 0.05). Piglets fed with LMR + S in the OPTIMAL hygiene had higher ADFI from day 4 to 28 and from day 11 to 28, TDMI, total energy intake, and total lysine intake than the LMR + S-fed piglets in the SUB-STANDARD hygiene and the DPS-fed piglets in both the OPTIMAL and SUB-STANDARD hygiene. During the entire pre-weaning period, each LMR + S-fed piglet in the OPTIMAL hygiene consumed 228 g of DM of milk replacer and 350 g of DM of starter diet, while each LMR + S-fed piglet in the SUB-STANDARD hygiene consumed 130 g of DM of milk replacer and 181 g of DM of starter diet on average.

There were no differences in pig bodyweight at day 4 between any treatment groups. On day 28, there was a creep feeding × hygiene interaction for bodyweight (*P* ≤ 0.05); piglets fed with LMR + S in the OPTIMAL hygiene had a higher bodyweight than DPS-fed piglets in both the OPTIMAL and SUB-STANDARD hygiene (*P* ≤ 0.05). On day 28, LMR + S-fed piglets in the SUB-STANDARD hygiene were heavier than DPS-fed piglets in the SUB-STANDARD hygiene (*P* ≤ 0.05). On day 28, there was no difference between the bodyweights of the LMR + S-fed or the DPS-fed piglets in the OPTIMAL vs SUB-STANDARD hygiene. There was no main effect of hygiene on bodyweight on day 28 but there was for creep feeding, with LMR + S-fed pigs heavier than DPS-fed pigs (*P* ≤ 0.05). There was no creep feeding × hygiene interaction or main effect of hygiene for ADG from day 4 to 28. However, there was a main effect of creep feeding where LMR + S-fed piglets had higher ADG compared to DPS-fed piglets (*P* ≤ 0.05). There was no effect of hygiene or creep feeding or their interaction on the CV% of within-litter piglet bodyweight at day 4. However, there was a creep feeding × hygiene interaction for this measure at day 28 (*P* ≤ 0.05), where LMR + S-fed pigs in the SUB-STANDARD hygiene had a lower CV% compared to DPS-fed pigs in the OPTIMAL hygiene. No other differences were observed for CV% on day 28.

### Live observation of feeding behavior

The effect of creep feeding regime, hygiene, and their associated interactions on creep feeding behavior of suckling piglets is presented in Supplementary Table S4. On day 6, a higher proportion of LMR + S-fed piglets in the OPTIMAL hygiene was observed to direct behavior toward the feeder than LMR + S-fed piglets in the SUB-STANDARD hygiene (*P* ≤ 0.05). On day 13, there was a creep feeding × hygiene interaction, whereby LMR + S-fed piglets in the SUB-STANDARD hygiene were observed to express a lower percentage of feeder-directed behavior than LMR + S-fed piglets in the OPTIMAL hygiene and DPS-fed piglets in the OPTIMAL hygiene (*P* ≤ 0.05). On day 13, there was no main effect of creep feeding but piglets reared in the OPTIMAL hygiene were observed to have a greater percentage of feeder-directed behavior than piglets reared in the SUB-STANDARD hygiene (*P* ≤ 0.05). On day 20, there was also a creep feeding × hygiene interaction, with LMR + S-fed piglets in the SUB-STANDARD hygiene observed to have a lower percentage of feeder-directed behavior than DPS-fed piglets in both the OPTIMAL and SUB-STANDARD hygiene (*P* ≤ 0.05). On day 20, there was no main effect of hygiene but DPS-fed piglets were observed to have a higher percentage of feeder-directed behavior than LMR + S-fed piglets (*P* ≤ 0.05). Finally, on day 27, there was again a creep feeding × hygiene interaction, where LMR + S-fed piglets in both the OPTIMAL and SUB-STANDARD hygiene were observed to have a lower percentage of feeder-directed behavior than DPS-fed piglets in both the OPTIMAL and SUB-STANDARD hygiene (*P* ≤ 0.05). On day 27, the LMR + S-fed piglets in the SUB-STANDARD hygiene were also observed to have a lower percentage of feeder-directed behavior than LMR + S-fed piglets in the OPTIMAL hygiene (*P* ≤ 0.05). On day 27, there was no hygiene main effect, but DPS-fed piglets were observed to have a higher percentage of feeder-directed behavior than LMR + S-fed piglets (*P* ≤ 0.05).

### Post-weaning growth performance and carcass data

There was no effect of hygiene or creep feeding regime or their associated interactions on ADFI, ADG, or G:F of pigs from weaning to slaughter or on carcass parameters (Table 4). Nonetheless, pigs originating from OPTIMAL hygiene farrowing pens reached target slaughter weight 3.8 days earlier than pigs originating from SUB-STANDARD hygiene farrowing pens (*P* ≤ 0.05). This is in line with the fact that feed intake and growth of pigs originating from OPTIMAL hygiene farrowing pens were numerically higher than that of pigs originating from SUB-STANDARD hygiene farrowing pens.

**Table 4.** Effect of creep feeding (DPS or LMR + S) and pre-farrowing hygiene routine (SUB-STANDARD or OPTIMAL) on feed intake, growth, gain to feed ratio, and carcass parameters of weaned pigs from weaning to slaughter [Least square means with their pooled standard errors of the mean (SEM)]

Creep feeding^1^	DPS		LMR + S			*P*-value		
Hygiene^1^	OPTIMAL	SUB-STANDARD	OPTIMAL	SUB-STANDARD	SEM	Hygiene	Creep feed	Hygiene × Creep feed
Number of pens	12	12	12	12				
Bodyweight, kg								
Day 0 (weaning)	8.4	8.5	9.4	8.5	0.43	0.33	0.27	0.23
Live weight at slaughter	130.3	130.0	130.0	129.3	2.12	0.59	0.80	0.92
ADFI^1^ from weaning to slaughter, g/pig/day	870	861	889	866	17.7	0.54	0.79	0.54
ADG^1^ from weaning to slaughter, g/pig/day	549	537	548	536	8.96	0.60	0.77	0.24
G:F^1^ from weaning to slaughter, g/g	0.76	0.75	0.76	0.75	0.01	0.73	0.63	0.49
Weaning to slaughter duration, days	130.1	133.0	127.2	132.4	0.60	0.03	0.38	0.44
Carcass parameters								
Cold weight, kg	99.6	99.7	99.5	99.3	1.52	0.84	0.83	0.52
Kill out, %	76.3	76.9	76.3	77.0	0.41	0.15	0.89	0.90
Lean meat, %	57.7	58.0	57.8	58.1	0.24	0.27	0.58	0.94
Muscle depth, mm	57.3	57.1	55.6	56.2	1.14	0.89	0.26	0.72
Fat depth, mm	15.0	14.6	14.6	14.3	0.30	0.27	0.26	0.92

^1^DPS = Suckling piglets provided with dry pelleted starter diet from day 11 to 28 of age; LMR + S = Suckling piglets provided with a liquid mixture of milk replacer and starter diet from day 4 to 28 of age; OPTIMAL = Suckling piglets born in farrowing accommodation cleaned with an optimal hygiene routine; SUB-STANDARD = Suckling piglets born in farrowing accommodation cleaned with a sub-standard hygiene routine; ADG = Average daily gain; ADFI = Average daily feed intake; G:F = gain to feed ratio.

Growth performance data between day 0 and 4 PW for the 40 euthanized pigs is presented in Supplementary Table S5. As their feed intake was recorded on a pen basis, statistical analysis could not be performed on their ADFI but OPTIMAL hygiene pigs had numerically higher feed intake than SUB-STANDARD hygiene pigs. There was no creep feeding × hygiene effect and no main effect of creep feeding regime for ADG of these euthanized pigs from day 0 to 4 PW. However, pigs originating from the OPTIMAL hygiene pens tended to have higher ADG from day 0 to 4 PW compared to those originating from the SUB-STANDARD hygiene pens (*P* = 0.08).

### Hematological parameters of weaned pigs

The effect of creep feeding regime and hygiene and their associated interactions on hematological parameters of weaned pigs on day 4 PW is presented in Table 5. There was a creep feeding × hygiene interaction for basophil percentage, where DPS-fed pigs from the SUB-STANDARD farrowing pens had a higher basophil percentage compared to DPS-fed pigs from the OPTIMAL farrowing pens (*P* ≤ 0.05), but there was no difference between LMR + S-fed pigs from SUB-STANDARD and OPTIMAL pens. There was a tendency for a creep feeding × hygiene interaction for the platelet count, with LMR + S-fed pigs from the OPTIMAL farrowing pens tending to have a lower platelet count than DPS-fed pigs from the OPTIMAL farrowing pens (*P* = 0.08). Pigs originating from the SUB-STANDARD hygiene farrowing pens had a higher eosinophil count (*P* ≤ 0.05) and tended to have a higher white blood cell count (*P* = 0.06), lymphocyte count (*P* = 0.07), monocyte count (*P* = 0.08), and eosinophil percentage (*P* = 0.08) than pigs originating from OPTIMAL hygiene farrowing pens. None of the other hematological parameters measured were impacted.

**Table 5.** Effect of creep feeding (DPS or LMR + S) and pre-farrowing hygiene routine (SUB-STANDARD or OPTIMAL) on hematological parameters of weaned pigs on day 4 post-weaning [Least square means ± pooled standard errors of the mean (SEM)]

Main effects of hygiene and creep feeding^1^	OPTIMAL	SUB-STANDARD	SEM for hygiene	DPS	LMR + S	SEM for creep feed	*P*-value		
							Hygiene	Creep feed	Hygiene × Creep feed
Number of pigs	20	18		19	19				
White blood cells, × 10^3^ cells/µL	5.24	7.16	0.69	6.74	5.66	0.69	0.06	0.27	0.76
Lymphocytes, × 10^3^ cells/µL	1.50	2.15	0.25	2.14	1.51	0.25	0.07	0.11	0.86
Lymphocytes, %	29.23	29.85	2.04	31.80	27.28	2.04	0.83	0.13	0.34
Monocytes, × 10^3^ cells/µL	0.43	0.58	0.06	0.56	0.45	0.06	0.08	0.18	0.35
Monocytes, %	8.65	8.57	0.85	8.50	8.72	0.85	0.95	0.85	0.59
Neutrophils, × 10^3^ cells/µL	3.21	4.28	0.47	3.90	3.58	0.47	0.12	0.63	0.88
Neutrophils, %	60.19	58.99	2.56	57.64	61.54	2.56	0.74	0.29	0.68
Eosinophils, × 10^3^ cells/µL	0.07	0.12	0.02	0.10	0.09	0.02	0.01	0.72	0.16
Eosinophils, %	1.38	1.94	0.22	1.47	1.85	0.22	0.08	0.24	0.34
Basophils, × 10^3^ cells/µL	0.03	0.04	0.01	0.04	0.03	0.01	0.45	0.39	0.30
Basophils, %	0.52	0.60	0.09	0.55	0.57	0.09	0.47	0.93	0.04
Red blood cells, × 10^6^ cells/µL	5.61	5.86	0.15	5.80	5.67	0.15	0.25	0.54	0.86
Hemoglobin, g/dL	10.05	10.36	0.26	10.31	10.09	0.26	0.40	0.56	0.52
Mean corpuscular volume, fL	60.07	60.13	0.57	60.31	59.86	0.57	0.93	0.61	0.35
Mean corpuscular hemoglobin, pg/cell	17.88	17.74	0.18	17.78	17.83	0.25	0.86	0.60	0.29
Mean corpuscular hemoglobin concentration, g/dL	29.77	29.50	0.20	29.5	29.77	0.20	0.36	0.36	0.76
Platelets, × 10^3^ cells/μL	191	232	38.3	187	237	36.9	0.47	0.35	0.08

^1^DPS = Suckling piglets provided with dry pelleted starter diet from day 11 to 28 of age; LMR + S = Suckling piglets provided with a liquid mixture of milk replacer and starter diet from day 4 to 28 of age; OPTIMAL = Suckling piglets born in farrowing accommodation cleaned with an optimal hygiene routine; SUB-STANDARD = Suckling piglets born in farrowing accommodation cleaned with a sub-standard hygiene routine.

### Fecal and intestinal microbiota

#### Fecal and intestinal microbiota diversity

**Figure 1.**
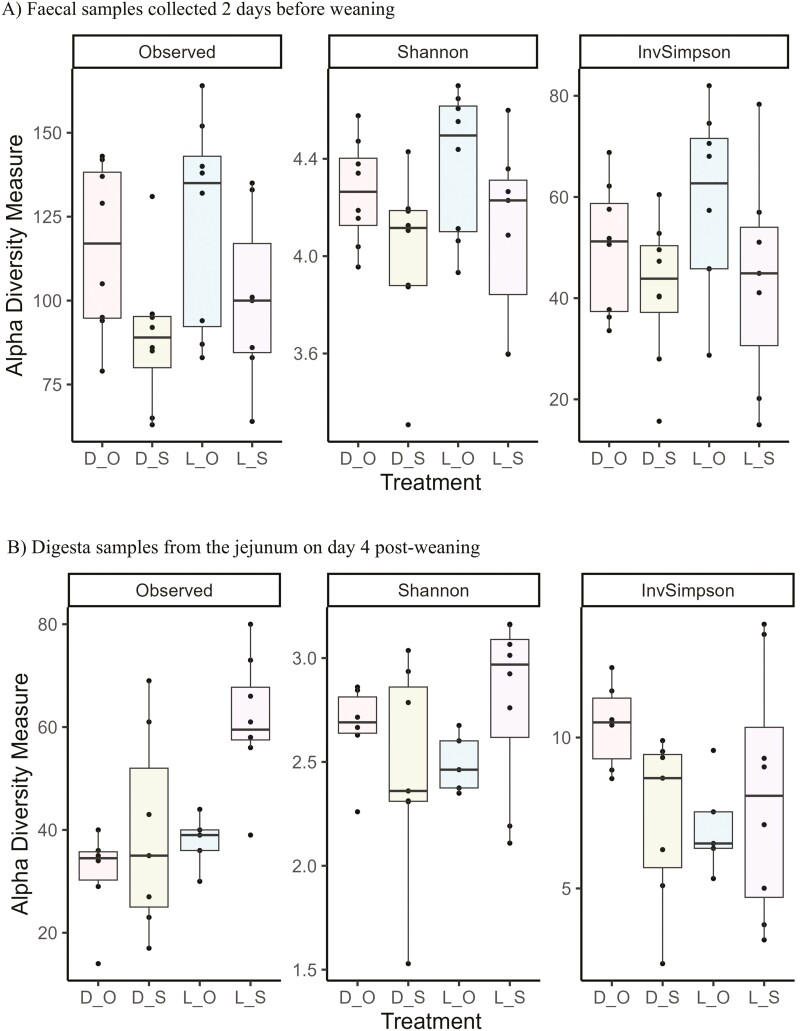

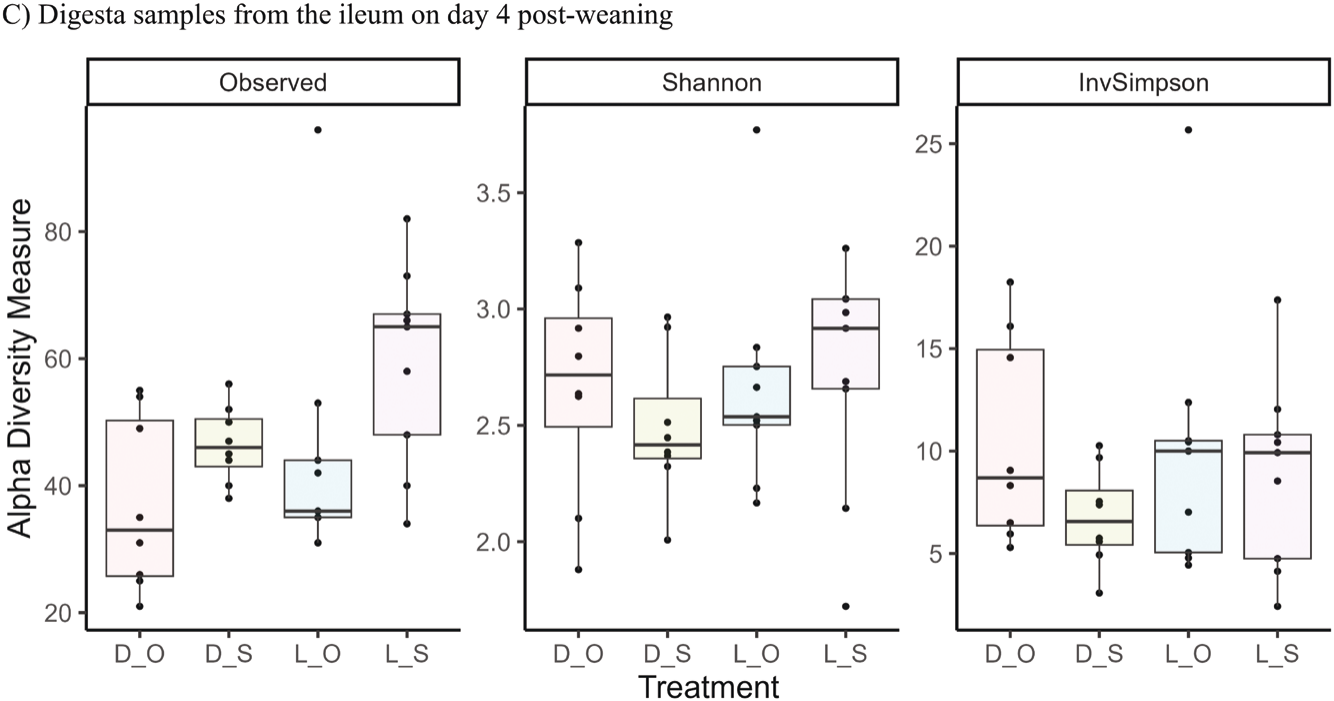
Effect of creep feeding (DPS or LMR + S) and pre-farrowing hygiene routine (SUB-STANDARD or OPTIMAL) on alpha diversity indices of fecal and intestinal microbiota of piglets, where D = Suckling piglets provided with dry pelleted starter diet from day 11 to 28 of age; L = Suckling piglets provided with a liquid mixture of milk replacer and starter diet from day 4 to 28 of age; O = Suckling piglets born in farrowing accommodation cleaned with an optimal hygiene routine; S = Suckling piglets born in farrowing accommodation cleaned with a sub-standard hygiene routine. (A) Fecal samples collected 2 days before weaning. (B) Digesta samples from the jejunum on day 4 post-weaning and (C) Digesta samples from the ileum on day 4 post-weaning.

**Figure 2.**
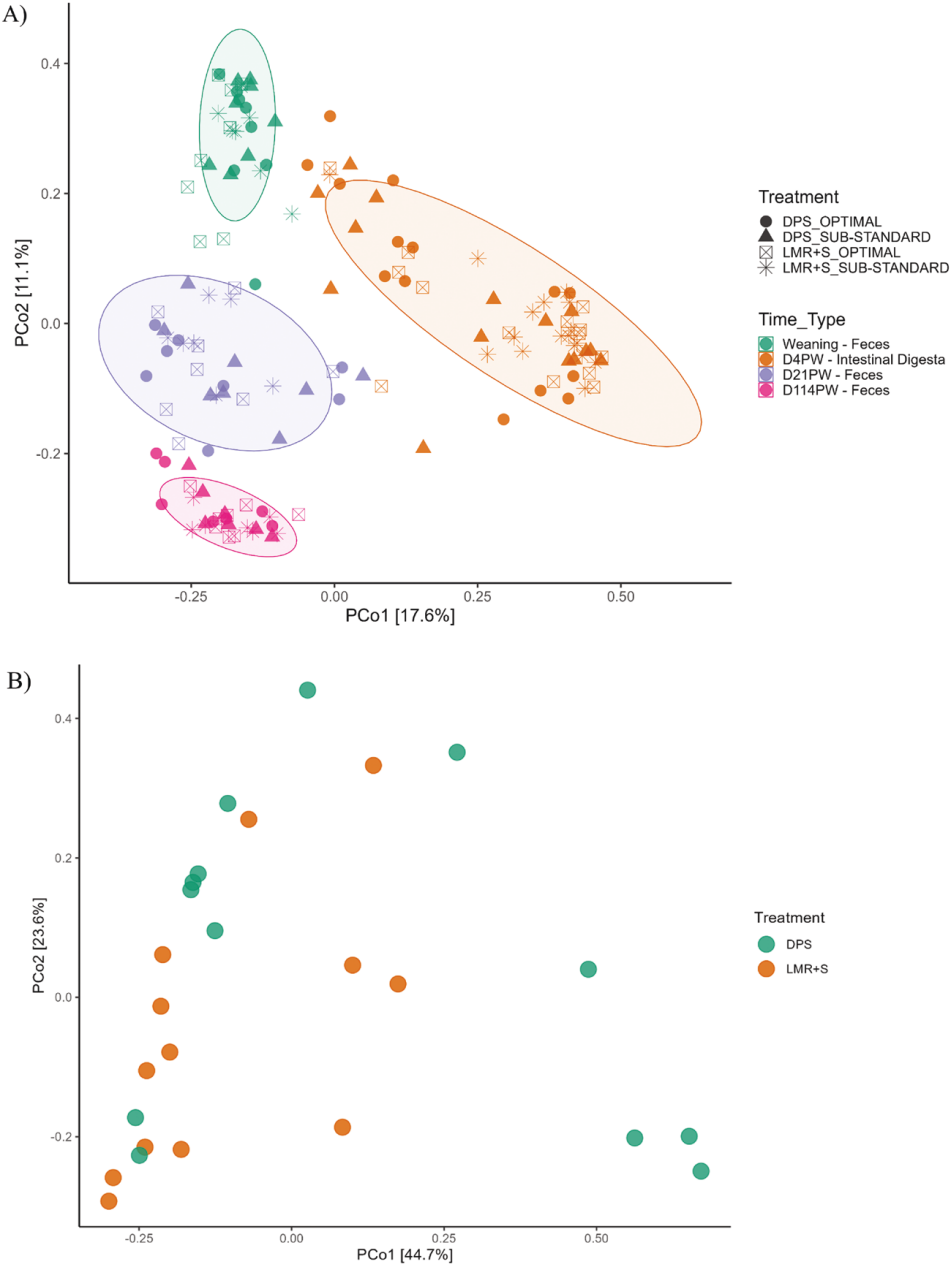
PCoA plot (β-diversity) of the fecal and digesta microbiota of pigs from all four treatments across all sampling time points. (A) Colors indicate the time point and sample type, i.e., digesta samples collected from the jejunum and ileum on day 4 post-weaning (D4PW) and fecal samples taken 2 days before weaning (Weaning), day 21 post-weaning (D21PW) and prior to slaughter at day 114 post-weaning (D114PW). Shapes indicate the treatments as follows: DPS = Suckling piglets provided with dry pelleted starter diet from day 11 to 28 of age; LMR + S = Suckling piglets provided with a liquid mixture of milk replacer and starter diet from day 4 to 28 of age; OPTIMAL = Suckling piglets born in farrowing accommodation cleaned with an optimal hygiene routine; SUB-STANDARD = Suckling piglets born in farrowing accommodation cleaned with a sub-standard hygiene routine. (B) Jejunum samples from DPS and LMR + S pigs on day 4 post-weaning.

There was no creep feeding × hygiene interaction for any of the alpha diversity indices at any sampling point in the fecal samples or in the jejunal and ileal digesta samples taken on day 4 PW. In the fecal samples collected 2 days prior to weaning (Weaning), there was no effect of creep feeding but there was an effect of hygiene (Figure 1A), with all three alpha diversity indices (observed, Shannon, and inverse-Simpson) higher in OPTIMAL than in SUB-STANDARD piglets (*P* ≤ 0.05). There were main effects of hygiene and creep feeding on the alpha diversity in the jejunum (Figure 1B) and ileum (Figure 1C) samples collected on day 4 PW; in both jejunum and ileum, the observed alpha diversity was higher in SUB-STANDARD than in OPTIMAL piglets (*P* ≤ 0.05) and in LMR + S than DPS piglets (*P* ≤ 0.05). The beta diversity of the fecal and digesta microbiota of all pigs across all sampling time points is represented using a PCoA plot (Figure 2A). In general, the fecal samples clustered according to sampling time point and the digesta samples clustered together. However, there was no distinct clustering according to treatment in any of the samples. There was only one treatment difference in beta diversity (Bray–Curtis distance), where the jejunal microbiota of the DPS and LMR + S groups differed (*P* ≤ 0.05), although no distinct clustering was observed (Figure 2B).

#### Fecal and intestinal digesta microbiota composition

**Figure 3.**
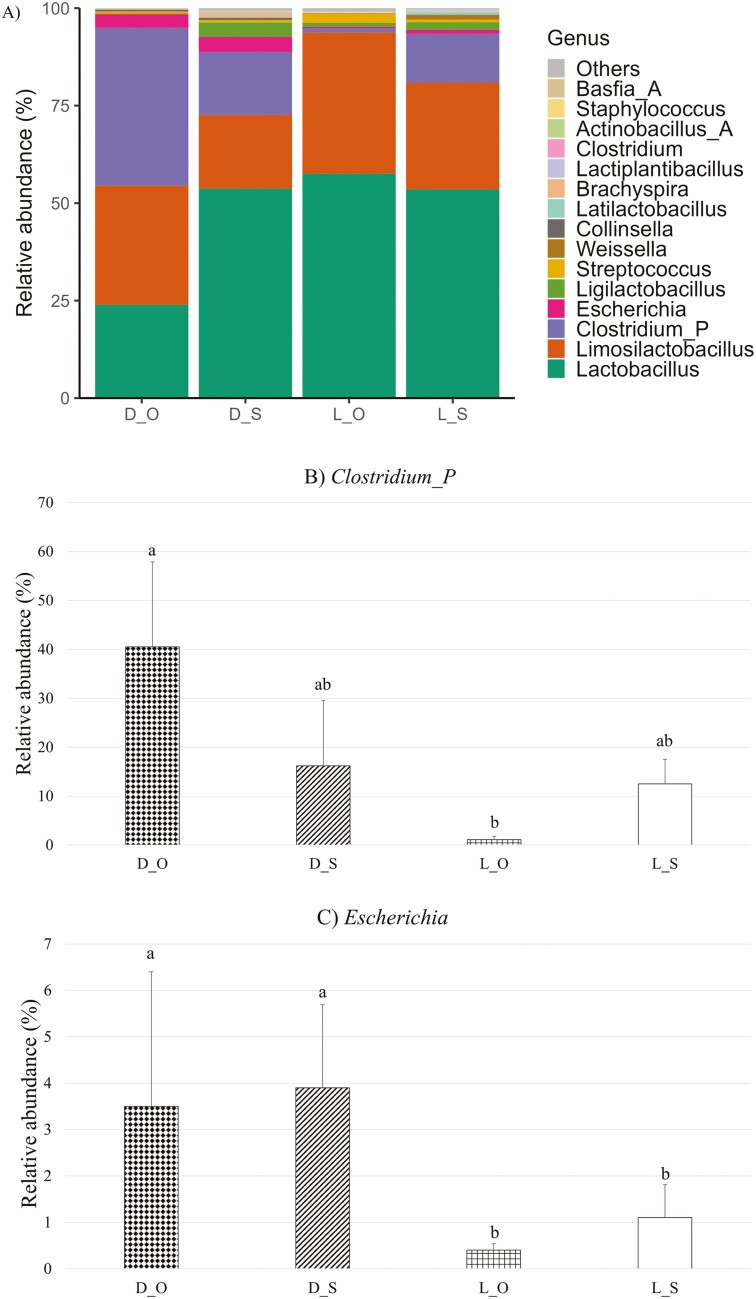
Effect of creep feeding (DPS or LMR + S) and pre-farrowing hygiene routine (SUB-STANDARD or OPTIMAL) on relative abundance (%) of bacteria genera in the jejunum digesta samples collected on day 4 post-weaning, where D = Suckling piglets provided with dry pelleted starter diet from day 11 to 28 of age; L = Suckling piglets provided with a liquid mixture of milk replacer and starter diet from day 4 to 28 of age; O = Suckling piglets born in farrowing accommodation cleaned with an optimal hygiene routine; S = Suckling piglets born in farrowing accommodation cleaned with a sub-standard hygiene routine. (A) Effect on mean relative abundance (%) of the 30 most abundant genera. (B) Effect on mean relative abundance (%) of the *Clostridium_P* genus. (C) Effect on mean relative abundance (%) of the *Escherichia* genus. a-b: Bars that do not share a common letter differ significantly at *P* ≤ 0.05.

**Figure 4.**
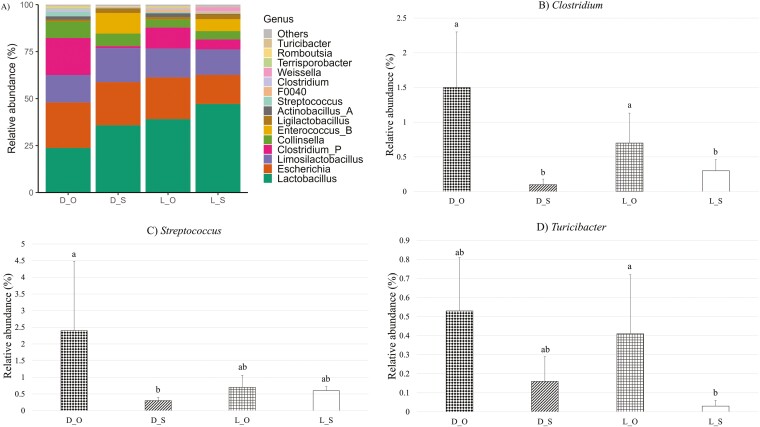
Effect of creep feeding (DPS or LMR + S) and pre-farrowing hygiene routine (SUB-STANDARD or OPTIMAL) on relative abundance (%) of bacteria genera in the ileum digesta samples collected on day 4 post-weaning, where D = Suckling piglets provided with dry pelleted starter diet from day 11 to 28 of age; L = Suckling piglets provided with a liquid mixture of milk replacer and starter diet from day 4 to 28 of age; O = Suckling piglets born in farrowing accommodation cleaned with an optimal hygiene routine; S = Suckling piglets born in farrowing accommodation cleaned with a sub-standard hygiene routine. (A) Effect on mean relative abundance (%) of the 30 most abundant genera. (B) Effect on mean relative abundance (%) of *Clostridium* genus. (C) Effect on mean relative abundance (%) of *Streptococcus* genus. (D) Effect on mean relative abundance (%) of *Turicibacter* genus. a-b: Bars that do not share a common letter differ significantly at *P* ≤ 0.05.

A total of 17 phyla, 76 families, and 221 genera were identified in the fecal and digesta samples across all sampling time points, with *Firmicutes* and *Bacteroidota* as the predominant phyla. The mean relative abundances of the 35 most abundant genera in all of the samples across all of the sampling time points are presented in a heatmap (Supplementary Figure S2). There was no effect of creep feeding or hygiene or their associated interactions on relative abundances of any of the genera detected in the fecal samples at any of the sampling time points. In the jejunal digesta, there was a creep feeding × hygiene interaction for the relative abundance of two genera; *Clostridium_P* and *Escherichia* (Figure 3). The relative abundance of *Clostridium_P* was higher in DPS-fed piglets from the OPTIMAL hygiene farrowing pens than in LMR + S-fed piglets from the OPTIMAL hygiene farrowing pens (*P *≤ 0.05). A BLAST search of the differentially abundant *Clostridium_P* sequence resulted in a best match with *Sarcina ventriculi*, which has been proposed to be re-named as *Clostridium ventriculi* (Lawson and Rainey, 2016). The relative abundance of *Escherichia* was higher in the jejunum of DPS-fed piglets from both the OPTIMAL and SUB-STANDARD hygiene farrowing pens than in the jejunum of LMR + S-fed piglets from both the OPTIMAL and SUB-STANDARD hygiene farrowing pens (*P* ≤ 0.05). A BLAST search of the differentially abundant *Escherichia* sequence resulted in a best match with *Escherichia coli* strain B20159 (but no information on the serotype of the strain was found). There were no effects of creep feeding or hygiene on relative abundance of any genera in the jejunal samples. In the ileal samples, there was a creep feeding × hygiene interaction for relative abundance of three genera: *Clostridium*, *Turicibacter*, and *Streptococcus* (Figure 4). The relative abundance of *Clostridium* was higher in the DPS- and LMR + S-fed piglets from the OPTIMAL hygiene farrowing pens compared to the DPS- and LMR + S-fed piglets from the SUB-STANDARD hygiene farrowing pens (*P *≤ 0.05). A BLAST search of the differentially abundant *Clostridium* sequence resulted in a best match with *Clostridium lentum*. The relative abundance of *Turicibacter* was higher in LMR + S-fed piglets from the OPTIMAL hygiene farrowing pens compared to LMR + S-fed piglets from the SUB-STANDARD hygiene farrowing pens (*P* ≤ 0.05). A BLAST search of the differentially abundant *Turicibacter* sequence resulted in a best match with *Turicibacter bilis*. The relative abundance of *Streptococcus* was higher in DPS-fed piglets from the OPTIMAL hygiene farrowing pens compared to DPS-fed piglets from the SUB-STANDARD hygiene farrowing pens (*P* ≤ 0.05). A BLAST search of the differentially abundant *Streptococcus* sequence resulted in a best match with *Streptococcus alactolyticus*. There were no main effects of creep feeding or hygiene on relative abundance of any genera in the ileum samples.

### Histological analysis of the small intestine

The effect of creep feeding regime and hygiene and their associated interactions on histological parameters in the duodenum, jejunum, and ileum of pigs on day 4 PW are presented in Table 6. There was no effect of creep feeding or hygiene or their associated interactions on duodenal VH, VW, CD, or VH:CD ratio and on jejunal VW or VH:CD ratio. However, there was a tendency for a creep feeding × hygiene interaction for VH in the jejunum, where DPS-fed piglets from the OPTIMAL pens tended to have greater VH compared to DPS-fed piglets from the SUB-STANDARD pens (*P* = 0.06). There was no creep feeding × hygiene interaction for CD in the jejunum. Jejunal VH and CD were not affected by creep feeding but were affected by hygiene (*P* ≤ 0.05); the OPTIMAL hygiene piglets had greater jejunal VH and jejunal CD than the SUB-STANDARD hygiene piglets. Ileal VH was not affected by creep feeding × hygiene interaction or the main effect of creep feeding. However, it was affected by hygiene, with the OPTIMAL hygiene piglets having greater ileal VH than the SUB-STANDARD hygiene piglets (*P *≤ 0.05). The ileal VW and VH:CD ratio were not affected by creep feeding × hygiene interaction or main effect of creep feeding but tended to be affected by hygiene; the OPTIMAL hygiene piglets tended to have wider villi (*P* = 0.07), and tended to have a higher VH:CD ratio than the SUB-STANDARD hygiene piglets (*P* = 0.08). There was no effect of creep feeding or hygiene or their associated interactions on ileal CD.

**Table 6.** Effect of creep feeding (DPS or LMR + S) and pre-farrowing hygiene routine (SUB-STANDARD or OPTIMAL) on small intestinal histology and brush border membrane enzyme activity (sucrase, maltase, and lactase) of piglets on day 4 post-weaning [Least square means with their pooled standard errors of the mean (SEM)]

Main effects of hygiene and creep feeding^1^	OPTIMAL	SUB-STANDARD	SEM for hygiene	DPS	LMR + S	SEM for creep feed	*P*-value		
							Hygiene	Creep feed	Hygiene × Creep feed
Number of pigs	20	20		20	20				
Villus height, µm									
Duodenum	297	290	9.1	299	288	9.1	0.61	0.38	0.42
Jejunum	280	246	9.3	264	262	9.1	0.02	0.83	0.06
Ileum	210	177	9.9	184	203	9.9	0.03	0.19	0.73
Villus width, µm									
Duodenum	139	130	4.9	134	135	4.9	0.20	0.91	0.38
Jejunum	118	116	3.6	117	118	3.6	0.68	0.85	0.91
Ileum	119	110	3.5	118	111	3.5	0.07	0.14	0.71
Crypt depth, µm									
Duodenum	227	224	10.4	223	228	10.4	0.80	0.69	0.12
Jejunum	210	187	7.8	200	196	7.8	0.05	0.71	0.19
Ileum	212	204	6.4	201	215	6.4	0.37	0.13	0.89
Villus height to crypt depth ratio, μm/μm									
Duodenum	1.26	1.33	0.05	1.32	1.28	0.05	0.29	0.54	0.86
Jejunum	1.37	1.33	0.05	1.34	1.36	0.05	0.55	0.80	0.83
Ileum	0.99	0.88	0.04	0.92	0.94	0.04	0.08	0.72	0.55
Sucrase, U/g									
Duodenum	0.38	0.30	0.05	0.36	0.32	0.05	0.27	0.49	0.85
Jejunum	2.10	2.79	0.41	2.39	2.50	0.41	0.25	0.85	0.39
Ileum	1.10	0.75	0.19	0.64	1.20	0.19	0.20	0.04	0.67
Maltase, U/g									
Duodenum	22.66	21.36	3.06	23.62	20.41	3.06	0.77	0.46	0.22
Jejunum	45.12	42.10	3.47	43.44	43.78	3.47	0.54	0.95	0.36
Ileum	28.87	27.90	5.07	25.22	31.55	5.07	0.89	0.38	0.60
Lactase, U/g									
Duodenum	4.85	6.63	0.97	6.38	5.10	0.97	0.20	0.36	0.72
Jejunum	14.42	15.94	2.30	14.23	16.13	2.30	0.64	0.56	0.58
Ileum	1.40	1.27	0.10	1.32	1.34	0.10	0.40	0.89	0.82

^1^DPS = Suckling piglets provided with dry pelleted starter diet from day 11 to 28 of age; LMR + S = Suckling piglets provided with a liquid mixture of milk replacer and starter diet from day 4 to 28 of age; OPTIMAL = Suckling piglets born in farrowing accommodation cleaned with an optimal hygiene routine; SUB-STANDARD = Suckling piglets born in farrowing accommodation cleaned with a sub-standard hygiene routine.

### Small intestinal brush-border membrane enzyme activity

The effect of creep feeding regime and hygiene and their associated interactions on brush-border membrane enzyme activity (sucrase, maltase, and lactase) in the duodenum, jejunum, and ileum of pigs on day 4 PW is presented in Table 6. Ileal sucrase activity, but not that in the jejunum and duodenum, was higher in LMR + S pigs than in DPS-fed pigs (*P* ≤ 0.05) but was not affected by creep feeding × hygiene interaction or by hygiene. Maltase and lactase activity in the three sampled regions was unaffected by creep feeding regime or by hygiene or their associated interactions.

## Discussion

### Effect of pre-farrowing hygiene routine on pig health and feed intake

The OPTIMAL cleaning protocol used in this study reduced bacterial counts in the farrowing pens, leading to the reduction in clinical cases of disease observed and the need for medication usage in suckling piglets. Implementation of the OPTIMAL cleaning protocol led to a considerable reduction in the prevalence of diarrhea, nearly halving the rates observed compared with that of the SUB-STANDARD cleaning protocol. Moreover, piglets originating from OPTIMAL hygiene farrowing pens had lower lymphocyte, monocyte, basophil, and eosinophil counts in blood indicating that these piglets experienced less immune stimulation (Le Floc’h et al., 2014). These results are in line with the findings from a previous study of ours, where bacterial counts in pen swabs, the number of clinical cases of disease, diarrhea prevalence, and medication usage were reduced using a similar pre-farrowing hygiene protocol (Halpin et al., 2024). Sows were washed and disinfected as part of the OPTIMAL cleaning protocol in both the previous and current studies. The previous study found no reduction in bacterial counts on the sows’ udder following washing and disinfection (Halpin et al., 2024). Although this was not measured, it is likely that this was the case also in the current study, which would indicate that sow washing and disinfection was not the most important step in the OPTIMAL cleaning protocol. Therefore, the reduction in bacterial load in the farrowing accommodation obtained by using the OPTIMAL hygiene routine was most likely the key driver behind the decreased occurrence of illness, immune stimulation, and need for medication in the current study. Suckling piglets are the second-most medically treated group of pigs, after weaned pigs (Dewulf et al., 2022), and therefore reducing antimicrobial usage during this production stage, as achieved in the current study, is an important outcome in the context of reducing the risk of antimicrobial resistance in pigs.

As hypothesized, piglet creep feed intake was increased in the OPTIMAL hygiene, especially for piglets provided with the LMR + S. Although, the feed intake of the DPS-fed piglets in the OPTIMAL and SUB-STANDARD hygiene was not different, DPS-fed piglets in the OPTIMAL hygiene had an 8% numerically higher TDMI. To our knowledge, this study is the first to investigate the effect of pre-farrowing hygiene routine on creep feed intake of suckling piglets and hence direct comparison with previous studies is not possible. However, hygiene was previously found to have similar effects in weaned piglets where poor hygiene resulted in reduced feed intake, growth, and feed efficiency (Le Floc’h et al., 2009; Kahindi et al., 2014). This was explained by increased occurrence of disease and the resultant increased antigen exposure, resulting in increased immune stimulation when hygiene is sub-standard (Le Floc’h et al., 2009; Kahindi et al., 2014). Both illness and immune stimulation can reduce feed-motivated behavior, leading to decreased feed intake (Johnson and von Borell, 1994; Dantzer, 2004). Moreover, nutrient utilization and metabolism by piglets is likely negatively affected, as energy is diverted toward raising an immune response and away from growth (Williams et al., 1997; Johnson, 1998; Pluske et al., 2018). Therefore, it was not surprising that poorer pig health and increased immune stimulation in the current study were associated with a reduction in feed intake and growth.

### Effect of creep feeding regime on feed intake, growth, and intestinal maturity

The effect of the OPTIMAL hygiene on feed intake was much more pronounced in LMR + S-fed piglets compared to those fed DPS, with feed intake almost doubled. Providing creep feed in liquid form likely contributed to this increase in feed intake. Martins et al. (2020) reported increased feed disappearance in gruel-fed compared to dry-fed suckling piglets. It is likely that creep feed provision in liquid form increases feed intake because piglets are more familiar with the liquid form (Patridge and Gill, 1993). Furthermore, the presence of milk replacer in the LMR + S creep feed might have contributed to the increase in voluntary feed intake of piglets. For example, the milk replacer contained high levels of lactose, which is highly palatable for piglets and leads to increased feed intake (Zhao et al., 2021). Moreover, the milk replacer used in this study contained porcine spray dried plasma, which is known to increase feed intake in weaned piglets (Van Dijk et al., 2001; Zijlstra et al., 2009) and possibly contributed to the increased creep feed intake in the present study. Of course, an obvious explanation for increased creep feed intake with LMR + S was the fact that it was provided 7 days earlier than the DPS. However, dry creep feeding is usually commenced in the second week after farrowing since there is a minimal benefit to creep feed intake from starting the practice earlier than this (Sulabo et al., 2010b; Yan et al., 2011) and as much as 80% of creep feed intake occurs in the last week of lactation (Tokach et al., 2020). Therefore, while it can be acknowledged that the differential in feeding duration between the two treatments may have increased DM intake of LMR + S pigs somewhat, we believe that this increase was minimal. Altogether, the combined benefit of liquid feeding and presence of milk replacer in the diet is most likely the reason for the increased feed intake of the LMR + S-fed pigs in OPTIMAL hygiene conditions.

As expected, the higher feed intake of LMR + S-fed piglets reared in the OPTIMAL hygiene led to an increased weaning weight compared to that found with DPS-fed piglets. Despite the starter diet having a similar energy content to milk replacer powder, piglets fed LMR + S and reared in the SUB-STANDARD hygiene were also heavier at weaning than those fed DPS, even though the TDMI was similar for both treatments. Another study from our group also found increased weight gain with creep feeding of liquid milk replacer compared to creep feeding a dry starter diet, although the DM intake of both diets was similar (Arnaud et al., 2024). The higher amount of lactose consumed by LMR + S compared to DPS-fed piglets may help to explain this outcome [the starter diet contained ~23% lactose, derived from whey permeate and skim milk powder (Sauvant et al., 2004), while the milk replacer contained ~41% lactose (Swinco, personal communication)]. Suckling piglets have a greater ability to digest lactose than plant-based carbohydrates, which are inadequately digested and absorbed (Zhao et al., 2021). This is due to the progression of intestinal enzyme activities, as lactase activity is high at birth and remains high for the first 2 to 3 weeks after birth, while sucrase and maltase activities are low during this period (Bailey et al., 1956; Manners, 1976; Aumaitre and Corring, 1978). The increased pre-weaning growth rate with LMR + S compared with DPS also likely contributed to the observed reduction in pre-weaning mortality. Overall, the importance of including milk replacer powder in liquid creep feeding strategies to maximize pre-weaning feed intake and growth and minimize mortality is highlighted by these findings.

Nonetheless, creep feed should contain plant-based ingredients in order to provide suckling piglets with early exposure to these ingredients to promote intestinal maturity and enzyme development, thereby facilitating the transition to a largely plant-based diet after weaning (Pluske et al., 1997; Tokach et al., 2020). In the current study, the higher creep feed (more exposure to plant-based ingredients) and energy intake resulting from LMR + S creep feeding increased sucrase activity on day 4 PW, thereby accelerating intestinal maturity of the piglets (Marion et al., 2005). Therefore, LMR + S can be considered an effective creep feeding strategy, as it increases feed intake and exposes piglets to plant-based ingredients, while incurring lower feed costs than creep feeding with milk replacer alone.

### Suckling piglet feeding behavior

Within-litter creep feed intake is usually highly variable, with the proportion of eaters of creep feed within each litter of pigs an important determinant of the success of creep feeding (Sulabo, 2009; Middelkoop, 2020; Tokach et al., 2020). In the current study, live observations of piglets engaging in feeder-directed activity were used to investigate the effect of treatment on the proportion of creep feed eaters within each litter. Although feeder-directed activity by piglets does not imply creep feed consumption *per se*, it does give an indication of the proportion of eaters within each pen. The effect of hygiene on the feeding behavior of piglets was clear throughout lactation. A higher percentage of piglets were observed engaging in LMR + S feeder-directed behavior in OPTIMAL pens compared to SUB-STANDARD pens, while the percentage of piglets engaging in DPS feeder-directed behavior was numerically higher in OPTIMAL pens compared to SUB-STANDARD pens. A higher percentage of piglets were observed to engage in DPS feeder-directed behavior than in LMR + S-feeder-directed behavior, but these results did not align with the feed intake findings. A possible explanation for this discrepancy could be that the LMR + S feeders were positioned to one side of the sow’s head at the front of the pen, whereas the DPS feeder (because it was bigger) was placed to one side of the sow in the middle of the pen. Due to the difference in feeder positioning and because the troughs for the LMR + S were semicircular as opposed to circular for the DPS, only three to four piglets could be observed engaging in LMR + S-feeder-directed activity at any one time, while six to seven piglets could engage with the DPS feeder at any one time. Furthermore, as the method of live observations involved instantaneous scan sampling, the duration of time spent by piglets at the feeder was not accounted for. Piglets might have spent less time at the LMR + S feeder, resulting in a lower number of observations of feeder engagement being recorded compared to DPS-fed piglets. The method of live observation for determining the proportion of eaters has been criticized in previous studies for not completely reflecting actual feed intake (Appleby et al., 1991) and not accounting for differences in ingestion rates (Wattanakul et al., 2005).

### Post-weaning intestinal structure

Disruption to intestinal structure, in terms of morphological changes to villi and crypts, is an indicator of early PW intestinal stress (Dong and Pluske, 2007). Reduced feed consumption in the days following weaning results in reduced VH and VW and a deepening of crypts, leading to a reduced VH:CD ratio. This happens because enterocytes lining the intestinal mucosa die due to inadequate energy and nutrient supply and due to the stress of weaning during the immediate PW period (Dong and Pluske, 2007). Disruption to intestinal morphology is associated with decreased digestive and absorptive capacity leading to reduced PW growth (Pluske et al., 1997). In the present study, the longer and wider villi of pigs reared in the OPTIMAL hygiene suggest that they experienced less intestinal damage at weaning (Dong and Pluske, 2007). Likewise, deeper crypts in these pigs indicate that increased cell proliferation was occurring in crypts to help maintain VH and absorptive area (Hedemann et al., 2003). Although deeper crypts for a prolonged time after weaning indicates a longer period of recovery of intestinal structure and is considered unfavorable, in the current study, deeper crypts in combination with longer villi on day 4 can be considered a positive effect as it suggests that the enterocytes are being replenished quickly after weaning. This effect of OPTIMAL hygiene on the morphology of the small intestine is likely due to the numerically higher feed intake of these pigs. Though direct comparison to farrowing accommodation hygiene studies is not possible due to the lack of literature, two previous studies in weaned piglets showed that pigs weaned into high hygiene pens have longer villi and deeper crypts compared to those in poor hygiene pens (Zhao et al., 2007; Cho et al., 2021). The authors attributed this to higher feed intake of the high hygiene piglets leading to increased nutrient availability for the enterocytes. This was also likely the case in the present study, as euthanized piglets from the OPTIMAL hygiene had numerically higher feed intake and tended to have better growth between weaning and day 4 PW.

### Intestinal microbiome

There were limited treatment effects on the fecal microbiome of pigs from weaning to slaughter, and the few differences observed around weaning were mainly driven by pre-weaning hygiene conditions. The pre-weaning fecal microbial community of OPTIMAL hygiene pigs was richer and more even compared to that of SUB-STANDARD hygiene pigs. Higher diversity microbial communities are generally more stable, resistant, and resilient (Shade et al., 2012). However, at day 4 PW the jejunal and ileal microbiota of SUB-STANDARD hygiene pigs was richer compared to that of OPTIMAL hygiene pigs. The differences observed between fecal and digesta microbial diversity are to be expected (García Viñado et al., 2024). The higher bacterial richness in the small intestine of SUB-STANDARD hygiene pigs suggests that there is greater competition for nutrients and energy between the microbiota and the host in these animals (Byndloss et al., 2018). This can have a negative impact on pig growth, as was the case in the SUB-STANDARD hygiene pigs. This theory is backed up by the fact that the opposite is also true, i.e., pigs supplemented with pharmacological levels of zinc oxide have lower intestinal microbial diversity but better growth performance (Shen et al., 2014; Crespo-Piazuelo et al., 2021).

Five bacterial genera in the digesta were impacted by treatment on day 4 PW but only those with a relative abundance > 1% in at least one treatment group are discussed here. The relative abundance of *Clostridium* and *Streptococcus* in the ileum was higher in pigs from the OPTIMAL compared to SUB-STANDARD hygiene. In the jejunum, *Escherichia* was more abundant in DPS- compared to LMR + S-fed pigs and *Clostridium_P* was present at lower abundance in LMR + S- than DPS-fed pigs from OPTIMAL hygiene. The BLAST search identified *C. lentum* as the best species match for the differentially abundant *Clostridium* sequence in the ileal digesta. While the *Clostridiaceae* family contains many species of relevance to pigs (Guo et al., 2020; Uzal et al., 2023), there is no information about the impact of *C. lentum*. The BLAST search of the *Clostridium_P* sequence that was differentially abundant in the jejunum identified the closest match as *S. ventriculi*, which has been proposed to be re-named as *Clostridium ventriculi* (Lawson and Rainey, 2016). *Clostridium/Sarcina ventriculi* has been associated with gastric ulcers in humans and abomasal bloats in ruminants (Edwards et al., 2008; Lam-Himlin et al., 2011). Although it is not clear if it causes gastric disorders in pigs, ileal abundance of *S. ventriculi* was previously reduced with pharmacological levels of dietary zinc oxide in pigs (Vahjen et al., 2011), suggesting that decreased small intestinal abundance of this bacterial species may be associated with better growth. Therefore, in the current study, the lower abundance of *C./S. ventriculi* in the LMR + S-fed pigs from OPTIMAL hygiene may to help explain the increased growth observed in these pigs. The genera *Streptococcus* and *Escherichia* contain many commensals, but also species/serotypes that are pathogenic to pigs (Luppi, 2017; Gottschalk and Segura, 2019). In the current study, the BLAST searches identified the closest matches of the *Streptococcus* and *Escherichia* sequences as *S. alactolyticus* and *E. coli* strain B20159, respectively. *Streptococcus alactolyticus* has previously been found in pig feces (Farrow et al., 1984; Robinson et al., 1988) and the *E. coli* strain (B20159) was isolated from seawater (Hooban et al., 2022). However, no information on either the pathogenicity or potential benefits of this species and strain is available. Therefore, the implications of the increased abundance of these *Streptococcus* and *Escherichia* sequences in OPTIMAL hygiene and DPS-fed pigs, respectively, in the current study are unknown.

### Post-weaning pig growth performance

After weaning, pigs from OPTIMAL hygiene farrowing pens had numerically higher growth rates, particularly during the finisher phase, resulting in these pigs reaching slaughter earlier than pigs from SUB-STANDARD hygiene farrowing pens. This indicates that pig producers can potentially save 3.8 days of finisher feed by implementing the OPTIMAL pre-farrowing hygiene routine. This is an economically important finding as the cost of implementing the OPTIMAL cleaning protocol can be offset by finisher feed cost savings. There was no impact of creep feeding regime on the feed intake, growth or feed efficiency of the pigs from weaning until slaughter, even following the increase in weaning weight achieved by creep feeding LMR + S in the OPTIMAL hygiene. Likewise, previous studies comparing different liquid creep feeding strategies with dry creep feeding did not observe a benefit of liquid creep feeding on PW growth performance (Martins et al., 2020; Christensen and Huber, 2021; Arnaud et al., 2024). One possible explanation for this could be that piglets have no experience of eating a dry pelleted diet (which is generally fed PW), when only fed liquid creep feed during lactation. Therefore, these piglets may temporarily struggle to adjust to consuming a dry pelleted diet after weaning, whereas that might not be the case if a diet in liquid form is provided after weaning.

## Conclusions

Implementing an optimal hygiene routine pre-farrowing reduced the bacterial load of the environment into which piglets were born and housed until weaning and reduced the prevalence of diarrhea and disease occurrence in pigs. Furthermore, pre-weaning creep feed intake was increased, intestinal structure in early PW was improved and weaning to slaughter duration was reduced when an optimal pre-farrowing hygiene routine was used. Creep feeding piglets with a liquid mixture of milk replacer and starter diet increased weaning weight, improved intestinal maturity, and reduced pre-weaning mortality compared to creep feeding with a DPS. Liquid creep-fed pigs originating from farrowing pens of optimal hygiene had lower relative abundance of *Clostridium_P* in the jejunum early PW, possibly contributing to the better growth observed in these pigs. Overall, rearing piglets in optimal hygiene conditions and implementing the liquid creep feeding strategy maximized DM intake and growth in suckling piglets.

## Supplementary Material

skae380_suppl_Supplementary_File

## Abbreviations

ASVs: amplicon sequence variants
ADFI: average daily feed intake
ADG: average daily gain
BLAST: Basic Local Alignment Search Tool
CFU: colony-forming units
CD: crypt depth
DM: dry matter
G:F: , gain to feed ratio
MRD: maximum recovery diluent
PW: post-weaning
TDMI: total dry matter intake
VH: villus height
VH:CD: villus height to crypt depth ratio
VW: villus width
