# Supplementary material for: Effect of pre-farrowing hygiene routine (sub-standard vs. optimal) and creep feeding regime (dry pelleted starter diet vs. liquid mixture of milk replacer and starter diet) on post-weaning intestinal parameters and growth to slaughter in pigs
Source: J Anim Sci. 2024 Dec 18;103:skae380. doi: 10.1093/jas/skae380 (PMC11781198; doi:10.1093/jas/skae380)
Supplement: skae380_suppl_Supplementary_File [file skae380_suppl_supplementary_file.docx]

Effect of **pre-farrowing hygiene routine** (sub-standard vs. optimal) and creep feeding regime (dry pelleted starter diet vs. liquid mixture of milk replacer and starter diet) on post-weaning intestinal parameters and growth to slaughter in pigs

Shiv R. Vasa*,^§^*,* Gillian E. Gardiner^§^, Paul Cormican^#^, Keelin O’Driscoll*, Giuseppe Bee ^†^, Peadar G. Lawlor^*^.

* Teagasc, Pig Development Department, Animal and Grassland Research and Innovation Centre, Moorepark, Fermoy, Co. Cork P61 C996, Ireland

*^#^* Teagasc, Animal and Grassland Research and Innovation Centre, Teagasc, Grange, Dunsany, Co. Meath, Ireland

^§^ Eco-Innovation Research Centre, Department of Science, South East Technological University, Waterford City, Co. Waterford X91 K0EK, Ireland

^†^ Swine Research Unit, Agroscope, Posieux 1725, Switzerland

Corresponding author: [Peadar.Lawlor@teagasc.ie](mailto:Peadar.Lawlor@teagasc.ie)

Supplementary tables

**Supplementary Table S1.** Proportion of milk replacer powder, water and dry pelleted starter diet on a fresh weight basis (grams) mixed and fed through the automated liquid feeding system

| **Ingredient** | **From day 4 to 9** | **From day 10 to 14** | **From day 15 to 17** | **From day 18 to 21** | **From day 22 to 28** |
| --- | --- | --- | --- | --- | --- |
| Milk replacer powder | 154 | 115.5 | 77 | 38.5 | 0 |
| Water | 846 | 846 | 846 | 846 | 846 |
| Dry pelleted starter diet | 0 | 38.5 | 77 | 115.5 | 154 |

**Supplementary Table S2.** Effect of creep feeding (DPS or LMR+S) and pre-farrowing hygiene routine (SUB**-**STANDARD or OPTIMAL) on body weight and back fat depth of sows [Least square means with their pooled standard errors of the mean (SEM)]

| **Creep feeding^1^** | **DPS** | | **LMR+S** | |  | **P-value** | | |
| --- | --- | --- | --- | --- | --- | --- | --- | --- |
| **Hygiene^1^** | **OPTIMAL** | **SUB-STANDARD** | **OPTIMAL** | **SUB-STANDARD** | **SEM** | **Hygiene** | **Creep feed** | **Hygiene × Creep feed** |
| Number of sows | 22 | 23 | 21 | 21 |  |  |  |  |
| Backfat depth, mm |  |  |  |  |  |  |  |  |
| Day 110 of gestation | 16 | 17 | 18 | 17 | 0.7 | 0.23 | 0.67 | 0.24 |
| Weaning^2^ | 14 | 13 | 14 | 13 | 0.8 | 0.19 | 0.98 | 0.71 |
| Change in back fat depth from day 110 to weaning^3^, mm | -3.3 | -4.2 | -3.1 | -4.5 | 0.8 | 0.19 | 0.98 | 0.71 |
| Bodyweight, kg |  |  |  |  |  |  |  |  |
| Day 110 of gestation | 277 | 276 | 288 | 276 | 6.6 | 0.32 | 0.41 | 0.38 |
| Post-farrowing^4^ | 237 | 238 | 243 | 244 | 3.5 | 0.85 | 0.23 | 0.95 |
| Weaning | 235 | 239 | 245 | 250 | 5.3 | 0.34 | 0.17 | 0.84 |
| Change in bodyweight from day 110 to weaning^5^, kg | -40 | -36 | -38 | -34 | 3.0 | 0.19 | 0.53 | 0.96 |
| Change in bodyweight from post-farrowing to weaning^6^, kg | -9.5 | -6.4 | -6.1 | -1.3 | 6.3 | 0.49 | 0.64 | 0.89 |

^1^ DPS = Suckling piglets provided with dry pelleted starter diet from day 11 to 28 of age; LMR+S = Suckling piglets provided with a liquid mixture of milk replacer and starter diet from day 4 to 28 of age; OPTIMAL = Suckling piglets born in farrowing accommodation cleaned with an optimal hygiene routine; SUB-STANDARD = Suckling piglets born in farrowing accommodation cleaned with a sub-standard hygiene routine.

^2^ Weaning = day 28 ± 1.2 of lactation.

^3^ Sow back fat depth change from day 110 to weaning = (sow back fat depth at weaning – sow back fat depth at day 110 of gestation).

^4^ Estimated value: empty farrowing weight = (sow weight at day 110 – (total born × 2.25)). The value of 2.25 kg is an estimate of the increased weight in the gravid uterus and in mammary tissue attributed to each pig in a litter (NRC, 1998).

^5^ Sow bodyweight change from day 110 to weaning = (sow bodyweight at weaning – sow bodyweight at day 110 of gestation).

^6^ Sow bodyweight change from post-farrowing to weaning = (sow bodyweight at weaning – sow bodyweight post-farrowing).

**Supplementary Table S3.** Effect of creep feeding regime (DPS or LMR+S) and pre-farrowing hygiene routine (SUB-STANDARD or OPTIMAL) on medication usage and prevalence of diarrhoea in weaned pigs [Least square means with their pooled standard errors of the mean (SEM)]

| **Main effects of hygiene and creep feeding^1^** | **OPTIMAL** | **SUB-STANDARD** | **SEM for hygiene** | **DPS** | **LMR+S** | **SEM for creep feed** | **P-value** | | |
| --- | --- | --- | --- | --- | --- | --- | --- | --- | --- |
|  |  |  |  |  |  |  | **Hygiene** | **Creep feed** | **Hygiene × Creep feed** |
| Number of pens | 24 | 24 |  | 24 | 24 |  |  |  |  |
| Antibiotic usage^2^, ml/pig/pen | 0.73 | 0.36 | 0.22 | 0.76 | 0.33 | 0.22 | 0.24 | 0.16 | 0.08 |
| Anti-inflammatory usage^2^, ml/pig/pen | 0.23 | 0.18 | 0.06 | 0.25 | 0.16 | 0.06 | 0.54 | 0.30 | 0.11 |
| Number of clinical cases, number/ pen^3^ | 1.29 | 1.17 | 0.29 | 1.42 | 1.04 | 0.29 | 0.36 | 0.76 | 0.09 |
| Prevalence of diarrhoea from day 0 to day 14 post-weaning^4^, % | 26.6 | 41.7 | 4.3 | 33.6 | 33.9 | 4.4 | 0.01 | 0.95 | 0.58 |

^1^ DPS = Suckling piglets provided with dry pelleted starter diet from day 11 to 28 of age; LMR+S = Suckling piglets provided with a liquid mixture of milk replacer and starter diet from day 4 to 28 of age; OPTIMAL = Suckling piglets born in farrowing accommodation cleaned with an optimal hygiene routine; SUB-STANDARD = Suckling piglets born in farrowing accommodation cleaned with a sub-standard hygiene routine.

^2^ The only antibiotic used was Unicillin (Procaine Benzylpenicillin, 300 mg/ml injection, Univet, Cootehill, Cavan, Ireland) and the only anti-inflammatory used was Loxicom 5 mg/ml injection, (Norbrook, Monaghan, Ireland). The values were averaged by dividing the total volume used per pen by the number of pigs in each pen.

^3^ The number of pigs in each pen that were treated on one or more occasion.

^4^ Visual scoring of fecal consistency at pen level was recorded on day 4, 7, 9 and 14 post-weaning using a 4-grade scoring system (Casey et al., 2007) as follows: 0 for dry pelleted feces; 1 for soft feces with shape; 2 for mild diarrhoea (very soft without shape or viscous liquid feces) and 3 for severe diarrhoea (watery or with blood). Prevalence of diarrhoea was calculated for each treatment by dividing the number of fecal score of 2 or 3 by the total number given from day 4 to 14 post-weaning and multiplying by 100.

**Supplementary Table S4.** Effect of creep feeding (DPS or LMR+S) and pre-farrowing hygiene routine (SUB-STANDARD or OPTIMAL) on creep feeding behaviour of suckling piglets [Least square means with their pooled standard errors of the mean (SEM)]

| **Creep feeding^1^** | **DPS** | | **LMR+S** | |  | **P-value** | | |
| --- | --- | --- | --- | --- | --- | --- | --- | --- |
| **Hygiene^1^** | **OPTIMAL** | **SUB-STANDARD** | **OPTIMAL** | **SUB-STANDARD** | **SEM** | **Hygiene** | **Creep feed** | **Hygiene × Creep feed** |
| Number of sows/litters | 17 | 17 | 16 | 16 |  |  |  |  |
| Piglets within pen observed to engage in feeder-directed behaviour^2^, % |  |  |  |  |  |  |  |  |
| Day 6 | - | - | 20^a^ | 8^b^ | 3.7 | 0.03 | - | - |
| Day 13 | 30^a^ | 22^ab^ | 31^a^ | 15^b^ | 3.8 | 0.01 | 0.37 | 0.02 |
| Day 20 | 46^a^ | 44^a^ | 32^ab^ | 18^b^ | 6.5 | 0.17 | 0.01 | 0.01 |
| Day 27 | 72^a^ | 71^a^ | 41^b^ | 22^c^ | 5.0 | 0.27 | <0.01 | <0.01 |

^1^ DPS = Suckling piglets provided with dry pelleted starter diet from day 11 to 28 of age; LMR+S = Suckling piglets provided with a liquid mixture of milk replacer and starter diet from day 4 to 28 of age; OPTIMAL = Suckling piglets born in farrowing accommodation cleaned with an optimal hygiene routine; SUB-STANDARD = Suckling piglets born in farrowing accommodation cleaned with a sub-standard hygiene routine.

^2^ The percentage of piglets observed to engage in feeder direct activity in each litter per observation day was calculated by dividing the number of piglets observed engaging in feeder direct activity at least once during the day with the litter size and multiplying by 100.

^a-c^ Values within a row that do not share a common superscript differ significantly at *P* ≤ 0.05.

**Supplementary Table S5.** Effect of creep feeding (DPS or LMR+S) and pre-farrowing hygiene routine (SUB-STANDARD or OPTIMAL) on numerical feed intake (statistical analysis not performed) and individual growth of the euthanized pigs between weaning and day 4 post-weaning [Least square means with their pooled standard errors of the mean (SEM)]

| **Creep feeding^1^** | **DPS** | | **LMR+S** | |  | **P-value** | | |
| --- | --- | --- | --- | --- | --- | --- | --- | --- |
| **Hygiene^1^** | **OPTIMAL** | **SUB-STANDARD** | **OPTIMAL** | **SUB-STANDARD** | **SEM** | **Hygiene** | **Creep feed** | **Hygiene × Creep feed** |
| Number of pigs | 10 | 10 | 10 | 10 |  |  |  |  |
| ADFI^1^ between weaning and day 4 post-weaning^2^, g/pig/day | 193 | 107 | 219 | 147 | - | - | - | - |
| ADG^1^ between weaning and day 4 post-weaning, g/pig/day | 218 | 69 | 168 | 135 | 50 | 0.08 | 0.87 | 0.25 |

^1^ DPS = Suckling piglets provided with dry pelleted starter diet from day 11 to 28 of age; LMR+S = Suckling piglets provided with a liquid mixture of milk replacer and starter diet from day 4 to 28 of age; OPTIMAL = Suckling piglets born in farrowing accommodation cleaned with an optimal hygiene routine; SUB-STANDARD = Suckling piglets born in farrowing accommodation cleaned with a sub-standard hygiene routine; ADG = Average daily gain; ADFI = Average daily feed intake.

^2^ Statistical analysis could not be performed on the ADFI between weaning and day 4 post-weaning, as feed intake was recorded on a pen basis.

Supplementary figures

**
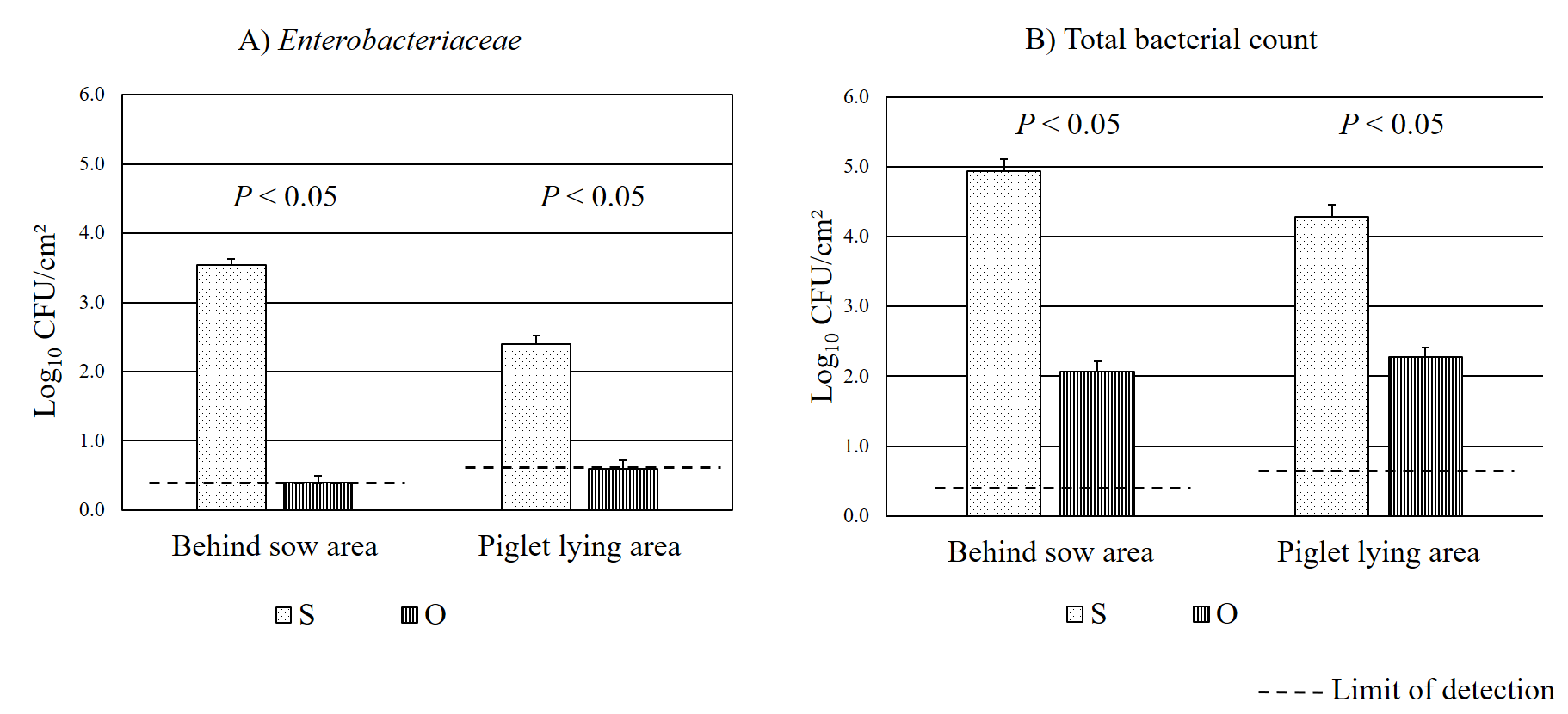
**

**Supplementary Figure S1.** Effect of pre-farrowing hygiene routine (SUB-STANDARD or OPTIMAL) on *Enterobacteriaceae* (A) and total bacterial counts (B) on pen floor areas (behind the sow and piglet lying area) before entry of the sows to the farrowing pens, where O = Optimal hygiene farrowing environment, and S = Sub-standard hygiene farrowing environment. Significant differences between treatments within each set of pen floor area samples are indicated as *P* < 0.05 and error bars represent the SEM. The limits of detection are indicated using a dashed line and values below the limit of detection were recorded as being at the limit of detection.

**
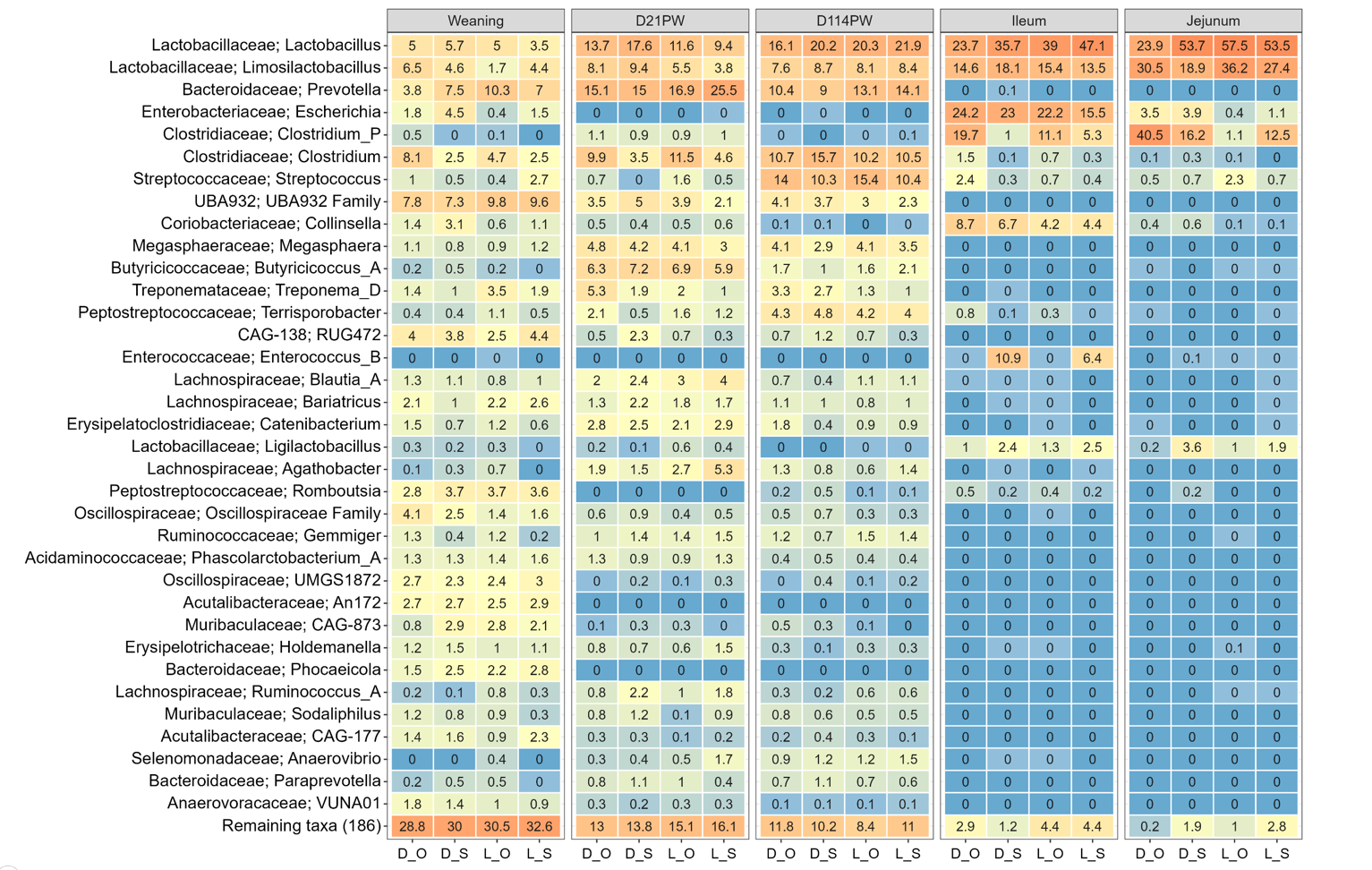
**

**Supplementary Figure S2.** Heatmap of the mean relative abundances of the 35 most abundant genera from all four treatments across all samples and sampling time points. Fecal sampling time points include two days before weaning (Weaning), day 21 post-weaning (D21PW) and prior to slaughter at day 114 post-weaning (D114PW). Digesta samples (jejunum and ileum) were collected on day 4 post-weaning. Treatments are: D = Suckling piglets provided with dry pelleted starter diet from day 11 to 28 of age; L = Suckling piglets provided with a liquid mixture of milk replacer and starter diet from day 4 to 28 of age; O = Suckling piglets born in farrowing accommodation cleaned with an optimal hygiene routine; S = Suckling piglets born in farrowing accommodation cleaned with a sub-standard hygiene routine.
